# Using iPSC models to examine neuron–glia interactions in neurodegenerative diseases

**DOI:** 10.1042/BSR20250131

**Published:** 2026-05-08

**Authors:** Dianne M. Lopez, Lois K. Keavey, Kathryn R. Bowles

**Affiliations:** 1 UK Dementia Research Institute at the University of Edinburgh, Edinburgh, EH16 4SB, Scotland, U.K.; 2 Institute for Neuroscience and Cardiovascular Research, University of Edinburgh, Chancellor’s Building, Edinburgh, EH16 4SB, Scotland, U.K.

**Keywords:** astrocytes, induced pluripotent stem cells, microglia, neurodegeneration, neurons

## Abstract

Neurodegenerative diseases remain without effective or accessible treatments and interventions, despite their increasing global burden. Clinically, these disorders are characterised by progressive cognitive decline, behavioural changes, and loss of motor function, all of which are associated with neuronal and synaptic loss or dysfunction. Although traditionally viewed as neuron-centric, it is becoming increasingly clear that glial cells play critical roles in maintaining and regulating neuronal and synaptic health. Mounting evidence implicates glial dysregulation in both the onset and progression of neurodegenerative diseases through mechanisms such as aberrant synaptic engulfment and protein clearance, impaired homeostatic support, metabolic dysfunction, chronic inflammation, transmission of pathogenic proteins, and cellular senescence. Elucidating how disruptions in neuron–glia interactions contribute to neuronal dysfunction is therefore essential for developing effective therapies. Induced pluripotent stem cell (iPSC)-based models provide a powerful platform to investigate these interactions in human-relevant systems. Here, we will discuss recent insights into the mechanisms contributing to neurodegenerative disease that have been gained specifically from modelling neuron–glia interactions in human iPSCs.

## Introduction

The increasing prevalence of neurodegenerative diseases, such as Alzheimer’s disease (AD), Parkinson’s disease (PD), and primary tauopathies, in an ageing population imposes a significant societal and economic burden due to their progressive nature, debilitating clinical outcomes and lack of any effective interventions or therapies. While each neurodegenerative disease has its own distinct clinical manifestation and neuropathological features, common hallmarks including protein misfolding and aggregation, synaptic dysfunction, metabolic impairment, and neuroinflammation are shared across conditions.

Although neurodegeneration is characterised by neuronal and synaptic loss, supporting glial cells are critical for maintaining neuronal health and homeostasis. While aberrant glial function has often been considered as a passive response to neuronal dysfunction, increasing evidence suggests that glia are actively involved in the pathogenesis and progression of neurodegenerative diseases [1,2], whether this be through aberrant engulfment of synapses [3,4], or dysfunction linked to chronic inflammation or senescence in later stages of disease [5–7]. Understanding the interplay between neurons and glia is therefore crucial to unravelling the mechanisms underlying disease progression and identifying potential therapeutic targets. As such, it is important that cellular functions are no longer considered only in individual isolated cell types but within the context of interactions with their environment and other cell types.

Induced pluripotent stem cell (iPSC) models have revolutionised study in this area, as they enable researchers to replicate specific cellular environments relevant to neurodegenerative diseases, offering insights into cell-specific phenotypes and precise modelling of cell–cell interactions, resulting in improved cellular accuracy for studying human disease [8–10]. Indeed, while animal models have been pivotal in uncovering disease mechanisms, translation of therapeutic interventions for neurodegeneration has so far been largely unsuccessful, likely due to significant evolutionary differences in brain structure, immune responses, and cellular interactions between species [11,12]. Despite valid criticism that iPSC models are limited with regard to their maturity and foetal-like state, they have consistently been shown to reproduce key disease-associated phenotypes in adult human brain [8,13–15] thereby supporting their validity and application to studying neurodegenerative disease.

In the present review, we summarise the current iPSC literature focused on neuron–glia interactions across multiple neurodegenerative diseases: AD, PD, and primary tauopathies, including progressive supranuclear palsy (PSP) and frontotemporal dementia (FTD-tau). While the present work emphasises the importance and relevance of neuron–glia interactions in the pathogenesis of neurodegenerative diseases, we also highlight critical areas in which additional insight is required and the potential for iPSC models to address these knowledge gaps.

## Alzheimer’s disease

AD is the most common neurodegenerative disorder [16–18], affecting almost a million people in the U.K. [19], with numbers expected to double in the next 20 years [20,21]. Pathologically, AD is characterised by the accumulation of extracellular amyloid-beta (Aβ) plaques and intracellular neurofibrillary tangles, composed of hyperphosphorylated tau protein. Amyloid pathology is thought to precede tau aggregation, with Aβ deposition beginning in the neocortical regions before spreading to deeper structures of the brain [22,23]. Tau pathology progresses in a stereotypical spatiotemporal pattern unique to each tauopathy. In AD, it emerges initially in the hippocampus and entorhinal region, spreading through the neocortex, limbic, and frontotemporal cortices, superolateral and occipital regions, as well as striatum and cerebellum. This progression, first described by Braak et al. [24], corelates with memory loss and cognitive decline. While significant neuronal loss underlies the clinical symptoms, numerous genetic and functional studies have highlighted critical roles for glial cells in AD risk, onset, and progression [25]. However, mechanisms by which glial dysfunction influences neuronal health and contributes to neurodegeneration are less well defined.

### Astrocytes

Astrocytes are the most abundant glial cells in the brain and are critical for regulating fundamental homeostatic processes that are important for the maintenance of neuronal health [26]. This includes production of antioxidants, regulation of neuronal signalling, extracellular ion balance, neurotransmitter metabolism, cytokine release, synapse modulation, and maintenance of the blood–brain barrier [26,27]. In addition, astrocytes exert neuroprotective functions by engulfing dead cells, clearing damaged synapses, and removing aggregated proteins [28,29]. In AD, both post-mortem and rodent models have demonstrated that astrocytic reactivity, neuroinflammation [27,30], mitochondrial and metabolic dysfunction [31], and the propagation of tau pathology [32] are closely linked to disease progression. Despite these advances, our understanding of neuron–astrocyte interactions in a human and disease-relevant context remains limited.

#### Inflammation

Growing evidence indicates that impaired degradation of pathogenic proteins and chronic inflammation by glial cells are key contributors to AD progression [27,33]. Specifically, aggregated tau and Aβ plaques have been shown to influence the reactive state of astrocytes and their ability to support neuronal health via both direct and indirect mechanisms [34–36]. For instance, neuron–astrocyte co-culture models have demonstrated that Aβ-exposed astrocytes disrupt neuronal excitatory post-synaptic currents and viability, releasing factors that alter neuronal intrinsic properties and network activity [28]. However, non-cell-autonomous dysfunction between neurons and astrocytes in AD is likely to be bidirectional. Activation of the adenosine receptor 1 (A1R) signalling pathway in iPSC-neurons from sporadic AD patients led to astrocytic activation and inflammation, which subsequently caused synaptic dysfunction in co-cultured healthy control neurons [37]. Pre-treatment of AD iPSC-neurons with the A1R antagonist, DPCPX, rescued these synaptic deficits, highlighting the reciprocal nature of neuron–astrocyte communication in the exacerbation of AD pathophysiology.

#### APOE genotype

**Figure 1:**
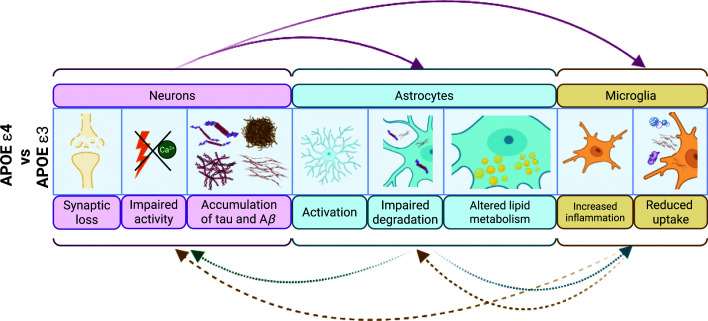
Differential effect of APOE genotype on neuron–glia interactions in health and disease *APOE Ɛ4* astrocytes show reduced uptake of Aβ_42_, increased cholesterol accumulation, and provide less support for neuronal survival and synaptogenesis compared with isogenic *Ɛ3* astrocytes, which are associated with reduced phosphorylated tau, Aβ aggregates, and synaptic loss. *APOE Ɛ4* microglia display up-regulated pro-inflammatory pathways and impaired phagocytosis of synaptosomes, myelin, and Aβ_42_, subsequently impairing neuronal calcium transients and network activity. Astrocytes and microglial cells carrying *APOE Ɛ3* exert neuroprotective effects by improving phagocytosis and clearance of pathogenic proteins, enhancing lipid metabolism, increasing neurotrophic support, and reducing pathogenic neuroinflammation compared with *APOE Ɛ4*.

Apolipoprotein E (APOE) is a cholesterol transporter protein that is primarily secreted by astrocytes. The human *APOE* gene has three main allelic variants: *Ɛ2, Ɛ3*, and *Ɛ4*, with *Ɛ4* associated with significantly increased risk of developing AD compared with the most common *Ɛ3* isoform [38]. This risk is likely to be due to both gain-of-toxic and loss-of-protective functions [39,40], although the precise molecular pathways underlying *APOE Ɛ4* pathogenicity are not yet fully elucidated. Regardless, the impact of *APOE* genotype appears to be broad; both *in vitro* and *in vivo* studies have reported that *APOE Ɛ4* contributes to impaired lipid metabolism, defective lipoprotein secretion, altered immune response, impaired Aβ clearance, and blood–brain-barrier disintegration [41–44] (Figure 1).

Isogenic sets of iPSC lines harbouring different *APOE* variants have been valuable in examining the specific effect of genotype in different cell types, such as neurons, astrocytes, and microglia, while maintaining the same genetic background [43,45]. In a study by Lin et al. [43], iPSC-astrocytes carrying *APOE Ɛ4* alleles display impaired lipid metabolism, reduced Aβ_42_ uptake and clearance, and increased cholesterol accumulation compared with their isogenic *Ɛ3* counterparts. Isogenic conversion of *APOE Ɛ4* (from sporadic AD donor iPSCs) to *Ɛ3* also attenuated AD-related phenotypes by reducing levels of Aβ aggregates, tau phosphorylation [43,46], apoptosis and synaptic loss [46]. Moreover, Zhao et al. [47] also showed that iPSC-astrocytes carrying *APOE Ɛ4* had impaired lipidation and were less able to support neuronal survival and synaptogenesis when co-cultured with neurons, compared with non-isogenic *APOE* Ɛ3 astrocytes. While the effect of *APOE* genotype clearly has distinct effects across different cell types, how exactly *APOE* expression in astrocytes impacts neuronal health has been less thoroughly examined.

The impact of *APOE* variants in astrocytes has also been explored in relation to the transmission of tau pathology [48–50]. Recent studies utilised iPSC models to examine the modifying effect of the rare protective *APOE* variant, *APOE Christchurch (APOE Ch)*, in the development of familial AD (fAD) symptoms in carriers of presenilin-1 (*PSEN1*) mutations [48]. Astrocytes carrying the *APOE Ɛ3Ch* variant were more resistant to reactivity upon exposure to inflammatory stimuli and suppressed propagation when co-cultured with *PSEN1 H163R* or *A246E* mutation neurons when compared with non-isogenic *APOE Ɛ3* astrocytes [48]. Interestingly, similar neuroprotective effects were observed in iPSC-microglia carrying the *APOE Ɛ3Ch* genotype when co-cultured with mutant neurons carrying *PSEN1* mutations [51].

#### Cell-to-cell spread of pathology

Astrocytes are increasingly recognised as active participants in cell-to-cell propagation of pathogenic proteins in AD [52]. Co-culture models demonstrate that iPSC-astrocytes actively internalise neuronally-derived tau [29] and Aβ [28,53] aggregates but fail to degrade them, subsequently secreting pathogenic materials into neighbouring healthy cells [54]. Neuron–astrocyte three-dimensional (3D) co-cultures further corroborate these findings, revealing efficient propagation of toxic tau oligomers across connected neuron–astrocyte 3D co-culture networks [55]. In the present model, human iPSC-neurons with tau oligomers were co-cultured with astrocytes to form spheroids (3D aggregates of one or more cell types that spontaneously assemble *in vitro*), which facilitated the spreading of AD-related phenotypes, including tau hyperphosphorylation, misfolding, and fibrillation, to both healthy neuronal networks and astrocytes. Single-cell transcriptomic and immunohistochemical analyses revealed significant transcriptional changes showing selective neuronal loss and astrocytic reactivity, accompanied by up-regulation of neuroinflammatory and stress-response genes, particularly the involvement of heat-shock protein (HSP) chaperone systems [55]. These transcriptional signatures mirrored those observed in post-mortem tauopathy brains assessed in the study [55]. Moreover, pharmacological inhibition of HSP90 reduced tau aggregation and astrocyte reactivity, thus highlighting the importance of the chaperone network in mediating the spread of tau pathology.

Uptake of pathogenic Aβ and tau aggregates may further compromise the neuroprotective functions of astrocytes by inducing inflammation and neuronal dysfunction [28,54]. Importantly, tau and Aβ species secreted by astrocytes exhibit a higher seeding capacity than neuron-derived aggregates, suggesting that astrocytic processing may exacerbate and amplify pathology [29,53]. Evidence also suggests that iPSC-astrocytes facilitate cell-to-cell propagation of pathogenic proteins via extracellular vesicles and tunnelling nanotubes (TNTs) [29,54], though these structures still require additional characterisation in neurons and astrocytes. However, this data suggests that proteostatic failure in astrocytes may facilitate protein accumulation and propagation, thereby sustaining and amplifying pathological spread. While both neurons and astrocytes secrete Aβ and tau [29,53,56], the exact mechanisms and non-cell-autonomous consequences of cell type-specific release have not yet been profiled.

#### Mitochondria dynamics and bioenergetics

Astrocytes are regulators of metabolism and mitochondrial function, processes which are disrupted in AD [57]. While bioenergetic disruptions observed in fAD, *presenilin-2* (*PSEN2*) and *amyloid precursor protein* (*APP*) mutation neuronal monocultures suggest a cell-autonomous contribution to metabolic dysfunction [58], co-culture models indicate a non-cell-autonomous effect of astrocytic metabolism dysfunction on neuronal health. For example, astrocytes derived from donors with the fAD *PSEN1ΔE9* mutation show deficits in lipid metabolism, increased Aβ production, increased oxidative stress and significant mitochondrial dysfunction, which consequently lead to disrupted calcium signalling activity in healthy control neurons [59,60]. Similarly, iPSC-neurons, -astrocytes, and -cerebral organoids derived from sporadic AD donors have each demonstrated alterations in mitochondrial dynamics [61,62] and metabolic dysfunction affecting fatty acid oxidation [57,59] compared with healthy controls.

Moreover, transmitophagy, which is the process mediating the intercellular transfer of mitochondria from neurons to astrocytes, followed by their degradation via mitophagy, appears to be impaired in *PSEN1*ΔE9 mutation models [63]. Specifically, *PSEN1*ΔE9 astrocytes exhibit a reduced capacity to process neuronal mitochondria compared with isogenic controls, further compromising neuron–glia metabolic coupling and mitochondrial control [63].

Collectively, these studies highlight multiple routes by which astrocytes influence neuronal health in the context of AD. These processes converge on aberrant inflammation, altered lipid metabolism, failure of proteostatic clearance, and impaired mitochondrial support as contributors to neuronal dysfunction.

### Microglia

Microglia are critical for regulating neuroinflammatory responses in the brain, with acute activation considered a protective response to neural damage [64,65], while chronic inflammation and microglial dysfunction are strongly associated with risk, onset, and progression of AD [17,65,66]. The function of microglial inflammation in the context of AD may therefore seem paradoxical; while microglia act protectively in the early stages of AD, they later actively contribute to neurodegeneration through aberrant phagocytic engulfment of neurons and synapses [67–69]. Microglia, including microglia–neuronal interactions, are currently widely studied in the context of AD, yet the majority of the present work has been conducted in rodent models and human post-mortem studies. Importantly, iPSC-microglia show similar secretory profiles to primary microglia in response to inflammatory stimuli and actively phagocytose a range of substrates, including Aβ and tau [70]. Furthermore, challenging iPSC-microglia with these substrates, as well as apoptotic neurons induces transcriptional changes towards a neurodegenerative disease-specific state, termed ‘damage-associated microglia’, based on expression of genes such as *APOE*, *GPNMB*, and *ABCA1* [71], indicating that, like in mice, iPSC-microglia have potential for faithfully modelling aspects of AD pathogenesis.

#### Inflammation

Cytokine and chemokine release by activated microglia have been hypothesised to contribute to the acceleration of AD progression by reducing phagocytosis and, therefore, extracellular Aβ clearance [72] and can be used to diagnose and monitor early-stage disease [73]. iPSC-microglia maintained in tri-culture with neurons and astrocytes have been shown to release significantly higher levels of complement component 3 (C3), a marker for inflammation associated with AD, following lipopolysaccharide exposure compared with microglia in monoculture, indicating that astrocytes and neurons can potentiate microglial C3 secretion [74]. Indeed, neurons carrying the double-point fAD Swedish mutation (*APP K670N, M671*L), which results in the overproduction of Aβ peptides, induced significantly higher C3 secretion from microglia than isogenic control neurons [74]. Tri-culture models leveraging 3D microfluidic platforms support the importance of the interplay between microglia, astrocytes, and neurons regulating neuroinflammation, microglial recruitment, and neurotoxic activities [75]. In these systems, separate interconnected microfluidic chambers allow precise control of cell positioning and communication through defined microchannels that permit only soluble factor exchange and directed cell migration. Park et al. developed a two-chamber microfluidic device to demonstrate interactions between iPSC-neurons and -astrocytes overexpressing fAD *APP* and *PSEN1* mutant proteins, plated within one microfluidic chamber, and immortalised Simian Virus 40 (SV40) microglia, cultured in an adjacent, angled chamber. Co-culturing these cells together led to an increase in microglial activation and migration and secretion of pro-inflammatory factors. Increased recruitment of reactive SV40 microglia caused further toxicity to AD neurons and astrocytes (e.g., elevated Aβ aggregation, tau phosphorylation and neuroinflammation) when compared with cultures in a non ‘AD environment’ [75]. This migration reflects chemotactic recruitment of microglia towards AD-associated pathological signals, modelling important *in vivo* cellular responses. Moreover, neuronal-microglial communication can be modified by *APOE* genotype: *APOE Ɛ4* microglia exhibit lipid accumulation and up-regulation of pro-inflammatory signals compared with *APOE Ɛ3* microglia [76]. To assess the functional impact of these microglia on neuronal networks, conditioned media from *APOE Ɛ4* microglia were applied to dissociated *APOE Ɛ3* neuronal spheroids, and neuronal activity was measured using a multielectrode array. Application of *APOE Ɛ4* microglia-conditioned media impaired the highly co-ordinated neuronal activity of *APOE Ɛ3* spheroids [76]. This indicates the importance of considering the cellular environment and interactions with other neural cell types for the regulation of microglial activity.

#### Neuronal and synaptic activity

When aberrantly activated, microglia may excessively prune synapses, leading to neuronal dysfunction and axonal injury [17], which has been shown to contribute to the onset and progression of neurodegeneration in rodent models [77–80]. However, while human *APOE Ɛ4* iPSC-microglia co-cultured with dissociated cortical spheroids impaired neuronal calcium transients and network activity in neurons, they had no effect on synaptic number or size [76] (Figure 1). In contrast, iPSC-microglia carrying the *TREM2 R47H* variant led to reduced synaptic density when xenotransplanted into mouse brains, despite displaying impaired uptake of synaptosomes, myelin, and Aβ_42_ *in vitro* [81]. These discrepancies may be due to *TREM2*-mediated up-regulation of complement and inflammation signalling in an *in vivo* context compared with *in vitro*, which may have either subsequently activated synaptic pruning [81] or induced neuronal dysfunction and synaptic loss via another mechanism. Alternatively, cross-species cellular interactions, or exposure to an adult brain environment, may have also influenced microglial activity and neuronal responses.

These inconsistencies highlight the need for a reproducible and robust tri-culture human-based system to investigate neuron–astrocyte–microglial cross-talk and how it contributes to disease pathogenesis [74,82,83]. These systems would also allow investigation of the interactions between astrocytes and microglia, which is an underexplored topic but likely to have significant effects in health and disease. For example, Lish et al. [82] recently reported significant transcriptomic and functional differences between mono- and tri-cultures consisting of iPSC-astrocytes, -neurons, and -microglia, including synaptic and neuronal organisation pathways, microglia reactivity, secretomes, and inflammatory signatures [82]. Moreover, iPSC-derived tri-culture models demonstrate that microglia are required for mediation of clusterin (CLU)-dependent *APOE* reduction in astrocytes, as well as increased levels of phosphorylated tau in neurons [82]. Loss of astrocytic CLU in this context caused microglia-dependent decreases in synaptic density (in tri-culture) and increased phagocytosis of exogenously applied neuronal synaptosomes (in microglia-astrocyte co-culture) [83]. Conversely, in tri-culture with fAD (*APP K670N, M671L*/*PSEN1 M146V)* neurons and *APOE Ɛ4* microglia, astrocytes with protective variants in *CLU* had increased *APOE* expression and conferred protection against the impact of fAD mutations by reducing levels of phosphorylated tau and increasing spine density in neurons [83].

## Parkinson’s disease

PD is the second most common and fastest-growing neurodegenerative disorder diagnosed among the ageing population in the world [84]. Globally, PD diagnosis is predicted to rise to 25.2 million people in 2050, representing an approximately 112% increase from 2021 [84]. PD patients clinically present with symptoms including rigidity, akinesia/bradykinesia, tremor, and postural instability [85]. Non-motor symptoms include cognitive impairments, hyposmia, depression, sleep disturbances, and autonomic dysfunction, which together with motor systems contribute to severe disability and reduced quality of life [85]. Pathologically, PD is characterised by the loss of dopaminergic neurons in the substantia nigra and intracellular accumulation of α-synuclein in the form of Lewy bodies and Lewy neurites [86]. The progression of pathology has also been proposed to be time-dependent and occurs in a region-specific manner, with pathogenic spread of α-synuclein, inflammation, and metabolic changes across different neural cell types all playing key roles [87–89].

**Figure 2:**
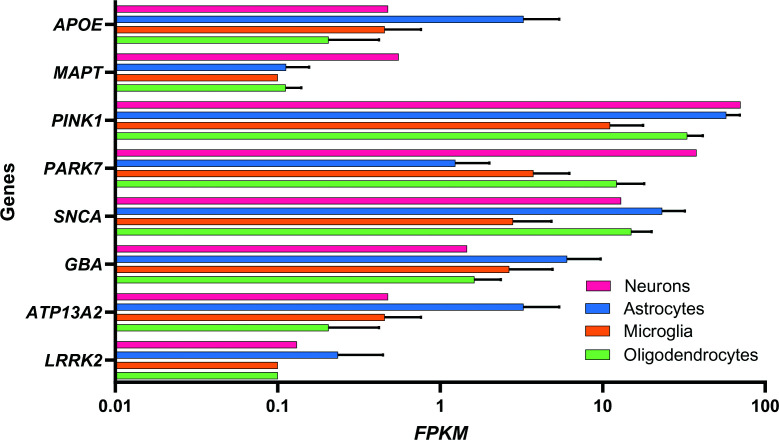
Differential expression of PD-related genes in astrocytes, neuron, microglia, and oligodendrocytes Transcriptomic data obtained from a publicly available human dataset reported by Zhang et al. [97]: human astrocytes (*n* = 12 subjects), neurons (*n* = 1), microglia (*n* = 3), and oligodendrocytes (*n* = 5). Gene expression is presented as fragments per kilobase of transcript per million mapped reads (FPKM). The graph shows mean ± SD. Generated through GraphPad.

While PD is characterised by the loss of specific neuronal populations, recent genetic studies of sporadic and familial forms of PD indicate involvement of genes that are highly expressed in glia, suggesting their involvement in disease pathogenesis [90–94]. Genome-wide association studies (GWAS) have been pivotal in identifying susceptibility loci, including leucine-rich repeat kinase 2 (*LRRK2*), glucocerebrosidase 1 (*GBA1*), α-synuclein *(SNCA)*, and the microtubule-associated protein tau (*MAPT*) locus, 17q21.31 [91,92,95,96]. Notably, the expression of PD-associated genes differs between rodents and humans [97] and also varies across distinct cell types in the human brain (Figure 2), underscoring the importance of human cellular models to complement and validate ongoing work in rodents [97,98].

### Astrocytes

Both rodent and iPSC models suggest that astrocytes play a role in the development of α-synuclein pathology and influence neuronal dopaminergic transmission and the release of neurotrophic factors, as well as antioxidant molecule production [99–101], suggesting that astrocyte health and function contribute to PD-related neuronal loss and the spread of pathology.

#### Accumulation and spread of pathology

A key hallmark of PD pathology is the intraneuronal accumulation of pathogenic α-synuclein-containing Lewy bodies/neurites, with reports of glial inclusions being rare [102]. Nevertheless, iPSC modelling of PD-related genes has provided insight into how astrocytes likely influence this process. Astrocytes may protect dopaminergic neurons from pathogenic protein accumulation and reduce α-synuclein propagation through neuronal networks by enhancing phagocytosis, endocytosis, and proteolytic capacity, thereby clearing extracellular α-synuclein [103]. Similar to their role in tau pathology, astrocytes are able to internalise pathogenic α-synuclein proteins released from degenerated or live neurons and send them for lysosomal degradation, thereby limiting its spread [103]. Moreover, astrocytes further provide support by releasing neurotrophic factors, producing antioxidants, regulating energy metabolism, and maintaining autophagy and chaperone-mediated degradation pathways [57,99,103]. In PD models, disruption to the astrocytic endolysosomal pathway specifically appears to be critical for promoting pathogenic α-synuclein spread. For example, mutations in astrocyte-expressed genes such as *ATP13A2*, which encodes a lysosomal type 5 P-type ATPase, impairs astrocytic phagocytosis, endocytosis, and proteolytic capacity, resulting in accelerated α-synuclein intracellular accumulation and intercellular propagation between neurons [103]. Furthermore, astrocytes harbouring risk-associated GBA1 mutations, a key gene responsible for regulating lysosomal function, show increased endocytosis of neuron-derived α-synuclein and translocation to dysfunctional lysosomes, leading to excessive α-synuclein accumulation, compared with isogenic controls [104].

Similar to processes previously described for tau and Aβ [28,29,53,54], inhibiting autophagic degradation of internalised material through activation of toll-like receptor 2 appears to further accelerate protein accumulation and spread [104,105], likely due to the inability of astrocytes to process and degrade pathogenic neuronal proteins, resulting in secretion and subsequently pathological spread. Di Domenico et al*.* [106] demonstrated that *LRRK2-G2019S* astrocytes exhibit dysfunctional chaperone-mediated autophagy (CMA) and impaired macroautophagy, leading to significant intracellular α-synuclein accumulation. Notably, pharmacological activation of CMA restored some functional α-synuclein degradation in these cells. Furthermore, *LRRK2-G2019S* astrocytes exerted a neurotoxic effect on dopaminergic neurons by facilitating the direct or indirect transfer and accumulation of astrocyte-derived α-synuclein in neurons, potentially promoting a cascade of pathogenic protein spread across connected neuronal networks [106]. When co-cultured with healthy neurons, *LRRK2-G2019S* astrocytes may also impair neurotrophic support by altering astrocyte-to-neuron shuttling of extracellular vesicles and by failing to secrete essential trophic factors and lipids, thereby contributing to neurodegeneration and reduced neurite development in dopaminergic neurons [106,107]. In contrast, co-culturing *LRRK2-G2019S* neurons with isogenic control astrocytes partially reverses mutation-associated phenotypes, resulting in improved neurite number and arborisation complexity [106]. Collectively, these findings highlight the impact of astrocytic functions in maintaining neuronal health and limiting α-synuclein pathology.

#### Mitochondria dynamics

Astrocytes regulate dopaminergic neuronal differentiation and maturation via modulation of mitochondrial dynamics [100]. Co-culturing iPSC-dopaminergic neurons with iPSC-astrocytes results in the reversal of mitochondrial morphological and dynamic defects induced by mitochondrial toxins [100], thus highlighting the capacity of astrocytes to support mitochondrial function and bioenergetics in the context of neuronal stress. In line with this, Cheng et al*.* [108] reported that astrocyte-derived healthy mitochondria could rescue dopaminergic neurons from degeneration caused by rotenone exposure, a commonly used mitochondrial toxin in PD models. Healthy astrocytes can spontaneously release their mitochondria into the extracellular space, which can then be internalised by injured neurons [108]. However, mitochondria derived from astrocytes with familial PD mutations are unlikely to provide the same neuronal support. For example, astrocytes carrying *PARK2* (encoding Parkin protein) mutations exhibit mitochondrial dysfunction and an increased release of pro-inflammatory cytokines, therefore impairing their ability to support dopaminergic neuron viability and function [109]. Astrocytic contributions to mitochondrial homeostasis therefore have the capacity to be either protective or detrimental to neuronal health, depending on the specific disease context or cellular environment.

Together, these studies show the dual role of astrocytes in the development and progression of PD. While they may have the potential to protect dopaminergic neurons by limiting α-synuclein propagation and supporting mitochondrial function in sporadic disease, familial mutations, such as *ATP13A2, GBA1, LRRK2*, and *PARK2*, are likely to impair the capacity for astrocytes to perform these protective functions, thereby exacerbating the pathogenic processes already underway in neurons.

### Microglia

Microglia play diverse roles in both physiological and pathological conditions, exhibiting both neurotoxic and neuroprotective effects. Physiologically, microglia are likely to be critical modulators of dopaminergic neuron differentiation and health; for example, co-culture of iPSC-derived human neural stem cells with microglia leads to a significant increase in dopaminergic neuron yield, which is accompanied by increased secretion of TNFα, IL-1β, and IGF1 by microglia and enhanced expression of key dopaminergic and midbrain-specific markers [110]. In the context of disease, microglia may play a similar role to astrocytes by facilitating the transmission of pathogenic α-synuclein, as well as promoting chronic neuroinflammation [111].

#### α-synuclein propagation

A major role for microglia is phagocytosis and subsequent degradation of aggregated proteins, like α-synuclein, as well as cellular debris [111]. Although study of the transmission of pathological α-synuclein has primarily been focused on neurons, there is growing consensus that microglia likely play a crucial role in its uptake, processing, and redistribution [111]. However, there is currently limited data from iPSC systems investigating neuron-microglia interplay in the context of PD, such that the way in which human microglia may respond to and process neuronally derived α-synuclein is yet to be assessed. Regardless, rodent studies indicate that microglia may contribute to pathological spread via TNTs [112] or exosome release [113–115]. These models also indicate that mouse microglia are unable to completely degrade phagocytosed extracellular α-synuclein, resulting in intracellular accumulation, followed by the induction of microglia reactivity and, subsequently, the exacerbation of α-synuclein propagation [111,114,116,117]. While these studies are valuable for profiling the contribution of microglia to PD pathology, given the functional and phenotypic differences that have been reported between human and rodent glial cells [117,118] (reviewed in [98,119,120]), replication in a human model system is still required.

#### Inflammation

Both genetic and environmental factors influence microglial inflammation observed in PD, including mutations in several PD-associated genes that result in cell-autonomous effects on microglial function and state [121–123]. For example, the *LRRK2-G2019S* mutation enhances phagocytic activity, dysregulates cytokine production, and increases microglial activation [122]. When co-cultured with neurons, mutant microglia induce neurite shortening and neurodegeneration, accompanied by impaired NF-κB p65 nuclear translocation and increased sensitivity of neurons to IFN-γ-mediated neurotoxicity [122]. This indicates that PD-associated microglial inflammation has the potential to amplify neuronal vulnerability to stressors, most likely resulting in a toxic feedback loop in which neuronal dysfunction further induces and sustains chronic microglial reactivity.

Consistent with this model, α-synuclein released from dopaminergic neurons carrying the *SNCA-A53T* mutation leads to NLRP3 inflammasome priming in co-cultured microglia [123]. This induced highly ramified microglial morphologies and increased secretion of pro-inflammatory cytokines such as IL-1β and TNF-β [123], which have also been reported in the brains and cerebral spinal fluid of PD patients [124]. Similarly, Blasco-Agell et al. [125] reported that neuromelanin, a by-product of dopamine metabolism that accumulates with age, can further drive neuroinflammation and neurotoxicity in dopaminergic neurons.

Data from post-mortem human brains support the assertion that glial reactivity is central to PD pathogenesis; single-cell transcriptomic analysis of human midbrain recently revealed a strong link between PD pathogenesis and glial activation, not only within microglia but also involving other glial cell types such as astrocytes and oligodendrocytes [126]. These findings suggest that, beyond neuronal dysfunction, broad inflammatory and stress-related responses across multiple cell types likely play a pivotal role in PD progression.

## Primary tauopathies

**Figure 3:**
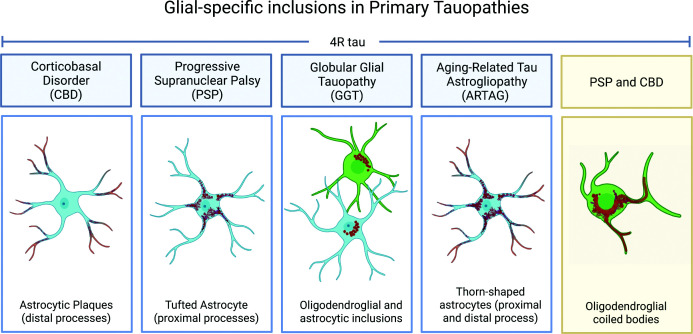
Cell-specific formation of tau inclusions in primary tauopathies Specific glial pathologies are observed across 4R tauopathies.

Primary tauopathies are a group of neurodegenerative disorders characterised by the accumulation of abnormally phosphorylated tau within neurons and glia, following disease-specific patterns of distribution (Figure 3) [127]. Clinically, primary tauopathies fall within the broad spectrum of frontotemporal lobar degeneration (FTLD) and include PSP and corticobasal degeneration, which are both considered sporadic primary tauopathies [128]. They occur with an estimated annual incidence rate of 2.4 and 0.4 per 100,000 people, respectively [127]. Around 11%–15% cases of FTLD are caused by pathogenic mutations in the *MAPT* gene, referred to as frontotemporal dementia with parkinsonism linked to chromosome 17 (FTDP-17) [129,130]. Primary tauopathies present with broad clinical manifestations, encompassing deficits in memory, speech, motor function, or behaviour, usually reflecting the specific brain regions and circuits affected [127].

In addition to their heterogeneous clinical presentations, primary tauopathies also demonstrate diverse cell-type involvement and distinct forms of neuropathological tau inclusions (Figure 3), as well as variability in the affected brain region and disease progression [127,131–133]. Neuropathologically, glial tau inclusions are characteristic of several primary tauopathies and, together with the regional distribution of tau pathology, serve as key post-mortem markers for distinguishing between different tauopathy subtypes. Regardless, little is currently known about how these glial pathologies contribute to disease pathogenesis.

### Astrocytes

Although pathological tau accumulates in astrocytes, it is not well understood how this pathology occurs, nor the subsequent impact on astrocytic function or neuronal health. As iPSC-astrocytes can exhibit functions and molecular hallmarks of *in vivo* astrocytes [15,134–137] and can further aid in the maturation and network development of iPSC-cortical neurons when co-cultured [138], these models could be highly valuable to address this question.

#### Neuronal activity and stress

Evidence from human studies and animal models have indicated the importance of bidirectional neuron–astrocyte interactions in cellular health and survival, synaptic expression, and tau pathology. Neuronal tau pathology may alter astrocyte–neuron interactions by promoting aberrant engulfment of synapses by astrocytes, as indicated by human *ex vivo* slice culture models of PSP [4]. Indeed, primary astrocytes derived from tauopathy mouse models (*MAPT-P301S* and *P301L*) negatively impact neuronal survival and reduce pre- and post-synaptic protein expression when co-cultured with wild-type (WT) primary neurons [139]. Furthermore, primary WT astrocyte transplantation into *MAPT-P301S* tauopathy mouse models reduces neuron loss [140], suggesting that even though *MAPT* expression is roughly 100-fold lower in astrocytes than neurons, it is sufficient to modulate astrocytic function and impact neuronal health in a non-cell-autonomous manner. Although currently limited in number, iPSC models have begun to replicate and extend aspects of these neuron–astrocyte interactions seen in post-mortem human and animal models; for example, iPSC-astrocytes carrying the *MAPT*-*IVS10+16* mutation have been shown to induce hyperexcitability in co-cultured isogenic control neurons. This effect was reversed when astrocytic *MAPT* expression was reduced with an antisense oligonucleotide [141], supporting the assertion that there are both cell-autonomous effects of *MAPT* mutation in astrocytes that subsequently have a non-cell-autonomous impact on neuronal function.

In contrast, neuronal dysfunction also significantly influences astrocytic health; overexpression of human *MAPT* with FTDP-17-associated *P301L* or *S320F* mutations resulted in tau aggregation in iPSC neurons, which subsequently induced oxidative stress and activation of the integrated stress response in co-cultured astrocytes [142]. Interestingly, exposing astrocytes to *MAPT*-mutation tau-containing neuron-conditioned media alone was not sufficient to induce this response, indicating direct interaction with dysfunctional neurons is necessary to induce astrocytic stress [142]. These iPSC models support the assertion that *MAPT* mutations within neurons and astrocytes have both cell-autonomous and non-cell-autonomous impacts on each other, resulting in modulated function and health of both cell types.

### Microglia

Tau pathology is linked to chronic neuroinflammatory processes, including both reactive astrocytosis and microgliosis [143,144]. Using new generation positron emission tomography ligands, such as 18 kDa translocator protein tracer 18 (F-GE-180), significant microglial activation has been measured in the brains of patients with 4R tauopathies [145]. As reported in models of AD, microglia may also play a role in tau pathology initiation and progression in primary tauopathies via abnormal phagocytosis of synapses [143,146,147]. Despite this, there are very few studies investigating microglia in the context of human tauopathy. Nevertheless, it has recently been reported that iPSC-microglia with the *MAPT*-*IVS10 + 16* mutation express both *MAPT* mRNA and tau protein, as do microglia derived from FTD-tau human brain [148]. Furthermore, this mutation is sufficient to induce cell-autonomous transcriptional changes in microglia related to phagocytosis and disease- and lipid-associated microglial states, as well as altered cytoskeletal networks and impaired phagocytosis of tau fibrils and myelin [148]. Much like astrocytes, low levels of endogenous *MAPT* expression, therefore, appears to have the capacity to influence microglial function and state, which is likely to have relevance for understanding the neuroinflammation associated with tauopathies. However, it remains to be examined how these microglial changes may influence neuronal health and function.

## Oligodendrocytes

Oligodendrocytes are the myelinating glial cells of the central nervous system and are essential for neuronal health and function by providing metabolic support and facilitating efficient signal transmission [149]. Although the interactions between neurons and oligodendrocytes are well-recognised in motor neuron diseases such as amyotrophic lateral sclerosis and neuropsychiatric disorders [150], their role in neurodegenerative diseases like AD, PD, and primary tauopathies remains underexplored, particularly in the context of human iPSC models.

Although iPSC models for neuron–oligodendrocyte interactions are limited, recent findings by single-cell transcriptomics and GWAS indicate that oligodendrocytes may contribute to the onset and progression of neurodegenerative diseases by negatively affecting myelination and influencing the aggregation of amyloid proteins [151–154]. Genes involved in stress [155], inflammation [156], and the unfolded protein response [126] have been reported to be altered in oligodendrocytes in the early stages of PD [153], while myelination abnormalities [126,156] and cholesterol metabolism, at a genetic and proteomic level [157,158], have also been reported to be altered in both PD and AD [159]. However, there is currently conflicting evidence regarding whether demyelination plays a role in PD, particularly in the substantia nigra [155,160]. While less thoroughly profiled, genetic and transcriptomic studies also implicate altered oligodendroglial function in primary tauopathies; for example, a recent PSP GWAS identified oligodendroglial expression of a novel locus (*C4a)* as associated with increased disease risk [161], and oligodendrocyte dysfunction, along with reduced myelin integrity, has been identified as a specific signature of *MAPT* mutation FTD subtypes [162]. Furthermore, oligodendrocytes are hypothesized to contribute to pathological tau spread [163], whereas the impact of *MAPT* mutations on neuronal microtubule stability may disrupt the local translation and myelin formation [164]. Collectively, these data indicate that oligodendrocyte function is likely to be highly relevant to the pathogenesis of multiple neurodegenerative diseases and warrants further investigation.

Considering the important role of oligodendrocytes in neuronal health and function, understanding their role in the development of neurodegenerative diseases such as AD, PD, and primary tauopathies and their interactions with other neural cells will be pivotal in understanding the cellular mechanisms underlying these diseases and developing new therapies. However, one of the major barriers to understanding neuron–oligodendrocyte interactions has been the difficulty in reliably and efficiently generating mature and myelinating oligodendrocytes from iPSCs. To address this, there are now several published protocols available that show promising results [165–167]. However, neuron-oligodendrocyte co-culture systems to model cellular interactions in both health and disease contexts still require further development and optimisation [168–170], yet recent co-culture system approaches show promise with more robust, stable, and time-efficient generation of mature neuron and oligodendrocyte populations from iPSCs [165]. Similar technologies have also been extended to generate organoids of myelinating oligodendrocytes [171,172], offering new ways to model neurodegeneration [168–170].

## Challenges and future directions

While iPSC models have numerous strengths with regard to modelling human neurodegenerative diseases, there are important limitations that should not be overlooked. A major hurdle is the maturity of differentiated cells, resulting in concerns surrounding how foetal-like cells could recapitulate adult, age-related disease. Direct reprogramming of donated human fibroblasts and other cell types *in vitro* is now being leveraged to generate neurons retaining age- and disease-related transcriptional profiles and molecular changes [45,173–177]. However, as described through the present review, it is worth noting that current foetal-like models are still able to successfully recapitulate relevant disease mechanisms and contribute valuable data even in the absence of ageing. Indeed, several groups have shown that both iPSC- and fibroblast-derived models are capable of reflecting disease-specific pathologies such as transactive response DNA-binding protein 43-related transcriptional changes [178] and accumulation of pathological tau [15,179], Aβ_1-40_ [179], and mutant huntingtin aggregates [180], amongst other cellular impacts [15,179–181].

In comparison with animal models, iPSC models have reduced biological complexity and cannot fully reflect pathogenic processes within an entire brain or organism. The advent of 3D brain organoids, first established by Lancaster et al. [182,183], is beginning to bridge this gap and is important for both overcoming the finite resource of human brain tissue and recapitulating the intricate architecture and microenvironment of human disease, including the interaction between numerous different neural cell types [184]. For example, *MAPT-*V337M mutant organoids were able to identify early cellular changes associated with FTD-tau, such as increased *MAPT* expression, altered glutamatergic signalling and autophagy pathways, loss of glutamatergic neurons, and production of stress granules, indicating the effects of this mutation precede neurodegeneration [185]. Human brain organoids have also provided better understanding of the association of AD risk genes and pathological characteristics associated with disease. Zhao et al. [46] have shown that *APOE ε4* is linked to severe synaptic dysfunction, elevated Aβ production, tau hyperphosphorylation, apoptosis, and stress granule formation but can be attenuated by conversion to *APOE ε3*. CRISPR/Cas9 knockout of the *APOE* gene in cerebral organoids has also shown that *APOE* deficiency has a large impact on neuronal differentiation, cholesterol biogenesis [186], lipid metabolism, and accumulation of insoluble α-synuclein [187].

Differentiation of brain region-specific 3D organoids has also provided opportunities for investigations within disease-relevant cellular environments. For example, midbrain organoids have been valuable for modelling PD and are able to recapitulate pathogenic mechanisms and impacts on neuron–glial interactions of *LRRK2-GS2019S* mutations, such as increased neurodegeneration, mitochondrial dysfunction, neurodevelopmental defects, increased α-synuclein aggregation, and defective protein clearance [188–190]. Midbrain organoids exhibit enriched neuronal development and astrocytic markers (e.g., *GFAP*, *S100β*, and *ALDH1L1* genes) when compared with 2D cultures, further highlighting the advantages of using 3D models in studying PD-related phenotypes [188]. Despite these advances, organoids are still not a complete model of human brain cell diversity, as they lack critical cell types and structures such as microglia, oligodendrocytes, or vasculature [191–193]. However, organoid models are amenable to the addition of these cell types to increase their complexity and functionality [194–198]. Fusion into ‘assembloids’ is also proving valuable to mimic more complex brain circuitry and connectivity, providing a network through which to investigate the spread of pathological proteins, axonal guidance alteration, and neuronal network function [199–201]. Yet, while organoid technology is advancing rapidly, it remains in its infancy. Widespread adaptation of microfluidic and organoid-on-chip platforms is already addressing key limitations such as the lack of vascularisation and the absence of molecular gradients that guide cell organisation and differentiation [202–204]. 3D microfluidic systems are also being used to model neuron–glia cross-talk to better understand disease pathogenesis in neurodegenerative diseases [75,205]. When coupled with artificial intelligence and sensitive biosensors [206], these technologies are expected to further accelerate investigations into neuron–glia interactions [205,207] and ultimately our understanding of the mechanisms underlying neurological and neurodegenerative diseases.

## Conclusion

iPSC models have been advantageous for studying neuron–glia interactions, as they allow precise examination of how neurons, astrocytes, microglia, and oligodendrocytes contribute to disease pathogenesis both alone and in defined, controlled combinations with other cell types. These models have clearly demonstrated that cellular responses are dependent on their surrounding environment, highlighting the relevance of considering cell–cell interactions and supporting the necessity for developing more complex models to reflect these intricacies.

Despite the vast knowledge and understanding into the mechanisms of neurodegenerative diseases that rodent studies have provided, it is important to consider that animal models do not completely recapitulate human disease and that neural cells, especially glia, have distinct transcriptional and functional differences between species [77,97]. In contrast, human iPSC models have been shown to recapitulate important aspects of neurodegenerative disease, spanning transcriptomic differences, inflammation, glial-mediated dysfunction, neuronal activity, and the spread of pathological proteins [15,46,71,148,179,180,189]. They provide the unique opportunity to model patient-specific conditions, as iPSC lines can be derived from individuals with early-onset, late-onset, or sporadic forms of neurodegenerative diseases and can facilitate personalised medicine by identifying patient-specific responses to therapeutic interventions. Furthermore, iPSC-derived systems have enabled high-throughput drug screening in a human model [184], which is valuable for determining human-specific cellular responses [208] and may aid in translational efforts for candidate therapeutics.

**Figure 4:**
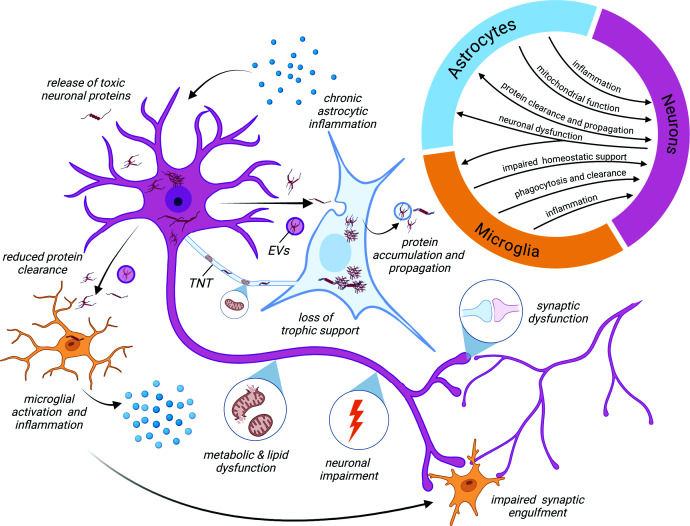
Common mechanisms of neuro–glia interactions contributing to neurodegenerative disease pathogenesis Astrocytic and microglial activation by neuronal dysfunction impacts neuronal trophic and metabolic support. Aberrant glia activity promotes synaptic dysfunction and engulfment, thereby impacting neuronal activity. The ability to clear pathological proteins such as tau, Aβ, and α-synuclein is reduced, while extracellular vesicles and TNTs promote pathological protein spread. Continued glial dysfunction further exacerbates neuronal impairment and drives neurodegeneration.

In the present review, we demonstrate that current work utilising co-culture and 3D iPSC models reveals that neuron–glial interactions in AD, PD, and primary tauopathies underlie a progressive, toxic bi-directional feed-forward cycle (Figure 4): neuronal dysfunction caused by intracellular protein dysregulation, protein transmission, and metabolic impairments may trigger a glial inflammatory response, which may be toxic to neuronal health. Equally, cell-autonomous dysfunction in either astrocytes or microglia (e.g., impaired protein clearance, mitochondrial impairment, lipid metabolism dysfunction, and inflammation) may either initiate or propagate a cascade of events leading to non-cell-autonomous impacts on neuronal health. Aberrant or chronic glial responses then serve to further disrupt neuronal health by either actively harming neurons through synaptic engulfment and neurite damage or passively failing to provide protection, as well as potentially promoting the transmission of pathological proteins around neuronal networks. Additional neuronal harm induces further glial activation, and the cycle continues. Although the initial triggers and exact sequence of events contributing to this cascade remain to be elucidated, advances in human iPSC modelling are now well-placed to be able to address these important questions and provide critical insight into the earliest mechanisms triggering and contributing to neurodegeneration.

## Open Access

Open access for this article was enabled by the participation of the University of Edinburgh in an all-inclusive Read & Publish agreement with Portland Press and the Biochemical Society under an agreement with JISC.

## Abbreviations

3D: three-dimensional
A1R: adenosine receptor 1
AD: Alzheimer’s disease
Aβ: amyloid-beta
APP: amyloid precursor protein
APOE: apolipoprotein E
CMA: chaperone-mediated autophagy
C3: complement component 3
fAD: familial AD
FTD-tau: frontotemporal dementia
FTLD: frontotemporal lobar degeneration
GWAS: genome-wide association studies
GBA1: glucocerebrosidase 1
HSP: heat-shock protein
iPSC: induced pluripotent stem cell
LRRK2: leucine-rich repeat kinase 2
MAPT: microtubule-associated protein tau
PD: Parkinson’s disease
PSP: progressive supranuclear palsy
SNCA: α-synuclein
SV40: Simian Virus 40
TNTs: tunnelling nanotubes
WT: wild-type

## References

[1] Kwon H.S. and Koh S.H. (2020) Neuroinflammation in neurodegenerative disorders: the roles of microglia and astrocytes. Transl. Neurodegener. 9, 1–12 10.1186/s40035-020-00221-2 33239064 PMC7689983

[2] Gleichman A.J. and Carmichael S.T. (2020) Glia in neurodegeneration: drivers of disease or along for the ride? Neurobiol. Dis. 142, 104957–104957 10.1016/j.nbd.2020.104957 32512150

[3] Briel N., Pratsch K., Roeber S., Arzberger T. and Herms J. (2020) Contribution of the astrocytic tau pathology to synapse loss in progressive supranuclear palsy and corticobasal degeneration. Brain Pathol. 31, e12914–e12914 10.1111/bpa.12914 33089580 PMC8412068

[4] McGeachan R.I., Keavey L., Simzer E.M., Chang Y.Y., Rose J.L., Spires-Jones M.P. et al. (2025) Evidence for trans-synaptic propagation of oligomeric tau in human progressive supranuclear palsy. Nat. Neurosci. 28, 1–13 10.1038/s41593-025-01992-5 PMC12321572 40670683

[5] Bellucci A., Westwood A.J., Ingram E., Casamenti F., Goedert M. and Spillantini M.G. (2004) Induction of inflammatory mediators and microglial activation in mice transgenic for mutant human P301S tau protein. Am. J. Pathol. 165, 1643–1643 10.1016/S0002-9440(10)63421-9 15509534 PMC1618683

[6] Olabarria M., Noristani H.N., Verkhratsky A. and Rodríguez J.J. (2010) Concomitant astroglial atrophy and astrogliosis in a triple transgenic animal model of Alzheimer’s disease. Glia 58, 831–838 10.1002/glia.20967 20140958

[7] Wang C., Fan L., Khawaja R.R., Liu B., Zhan L., Kodama L. et al. (2022) Microglial NF-κB drives tau spreading and toxicity in a mouse model of tauopathy. Nat. Commun. 13, 1–19 10.1038/s41467-022-29552-6 35413950 PMC9005658

[8] Valadez-Barba V., Cota-Coronado A., Hern Andez-P Erez O.R., Lugo-Fabres P.H., Padilla-Camberos E., An Díaz F. et al. (2020) iPSC for modeling neurodegenerative disorders. Regen. Ther. 15, 332–339 10.1016/j.reth.2020.11.006 33426236 PMC7770414

[9] Oberheim N.A., Wang X., Goldman S. and Nedergaard M. (2006) Astrocytic complexity distinguishes the human brain. Trends Neurosci. 29, 547–553 10.1016/j.tins.2006.08.004 16938356

[10] Li J. and Fraenkel E. (2021) Phenotyping neurodegeneration in human iPSCs. Ann. Rev. Biomed. Data Sci. 4, 83–83 10.1146/annurev-biodatasci-092820-025214 34465166 PMC9237961

[11] Burns T.C., Li M.D., Mehta S., Awad A.J. and Morgan A.A. (2015) Mouse models rarely mimic the transcriptome of human neurodegenerative diseases: a systematic bioinformatics-based critique of preclinical models. Eur. J. Pharmacol. 759, 101–117 10.1016/j.ejphar.2015.03.021 25814260

[12] Dawson T.M., Golde T.E. and Lagier-Tourenne C. (2018) Animal models of neurodegenerative diseases. Nat. Neurosci. 21, 1370–1370 10.1038/s41593-018-0236-8 30250265 PMC6615039

[13] Chang C.Y., Ting H.C., Liu C.A., Su H.L., Chiou T.W., Lin S.Z. et al. (2020) Induced pluripotent stem cell (iPSC)-based neurodegenerative disease models for phenotype recapitulation and drug screening. Molecules 25, 2000–2000 10.3390/molecules25082000 32344649 PMC7221979

[14] Li J., Pan L., Pembroke W.G., Rexach J.E., Godoy M.I., Condro M.C. et al. (2021) Conservation and divergence of vulnerability and responses to stressors between human and mouse astrocytes. Nat. Commun. 12, 3958 10.1038/s41467-021-24232-3 34172753 PMC8233314

[15] Bowles K.R., Pedicone C., Pugh D.A., Oja L.M., Sousa F.H., Keavey L.K. et al. (2024) Development of MAPT S305 mutation human iPSC lines exhibiting elevated 4R tau expression and functional alterations in neurons and astrocytes. Cell Rep. 43, 115013 10.1016/j.celrep.2024.115013 39602304 PMC13478741

[16] Deture M.A. and Dickson D.W. (2019) The neuropathological diagnosis of Alzheimer’s disease. Mol. Neurodegener. 14, 1–18 10.1186/s13024-019-0333-5 31375134 PMC6679484

[17] Leng F. and Edison P. (2020) Neuroinflammation and microglial activation in Alzheimer disease: where do we go from here? Nat. Rev. Neurol. 17, 157–172 10.1038/s41582-020-00435-y 33318676

[18] Li X., Feng X., Sun X., Hou N., Han F. and Liu Y. (2022) Global, regional, and national burden of Alzheimer’s disease and other dementias, 1990–2019. Front. Aging Neurosci. 14, 937486–937486 10.3389/fnagi.2022.937486 36299608 PMC9588915

[19] Local dementia statistics. Alzheimer’s Society. [Accessed 18 July 2025]. Available from: https://www.alzheimers.org.uk/about-us/policy-and-influencing/local-dementia-statistics

[20] Ittner L.M. and Götz J. (2011) Amyloid-β and tau—a toxic pas de deux in Alzheimer’s disease. Nat. Rev. Neurosci. 12, 67–72 10.1038/nrn2967 21193853

[21] Zhang X., Guo T., Zhang Y., Jiao M., Ji L., Dong Z. et al. (2024) Global burden of Alzheimer’s disease and other dementias attributed to metabolic risks from 1990 to 2021: results from the global burden of disease study 2021. BMC Psychiatry 24, 1–11 39696219 10.1186/s12888-024-06375-x PMC11654253

[22] Hampel H., Hardy J., Blennow K., Chen C., Perry G., Kim S.H. et al. (2021) The amyloid-β pathway in Alzheimer’s disease. Mol. Psychiatry 26, 5481–5481 10.1038/s41380-021-01249-0 34456336 PMC8758495

[23] Thal D.R., Rüb U., Orantes M. and Braak H. (2002) Phases of Aβ-deposition in the human brain and its relevance for the development of AD. Neurology 58, 1791–1800 10.1212/WNL.58.12.1791 12084879

[24] Braak H., Alafuzoff I., Arzberger T., Kretzschmar H. and Tredici K. (2006) Staging of Alzheimer disease-associated neurofibrillary pathology using paraffin sections and immunocytochemistry. Acta Neuropathol. (Berl) 112, 389–389 10.1007/s00401-006-0127-z 16906426 PMC3906709

[25] Nordengen K., Kirsebom B.E., Henjum K., Selnes P., Gísladóttir B., Wettergreen M. et al. (2019) Glial activation and inflammation along the Alzheimer’s disease continuum. J. Neuroinflammation 16, 1–13 10.1186/s12974-019-1399-2 30791945 PMC6383268

[26] Li K., Li J., Zheng J. and Qin S. (2019) Reactive astrocytes in neurodegenerative diseases. Aging Dis. 10, 664–675 10.14336/AD.2018.0720 31165009 PMC6538217

[27] Al-Ghraiybah N.F., Wang J., Alkhalifa A.E., Roberts A.B., Raj R., Yang E. et al. (2022) Glial cell-mediated neuroinflammation in Alzheimer’s disease. Int. J. Mol. Sci. 23, 10572 10.3390/ijms231810572 36142483 PMC9502483

[28] Konstantinidis E., Portal B., Mothes T., Beretta C., Lindskog M. and Erlandsson A. (2023) Intracellular deposits of amyloid-beta influence the ability of human iPSC-derived astrocytes to support neuronal function. J. Neuroinflammation 20, 3 10.1186/s12974-022-02687-5 36593462 PMC9809017

[29] Mothes T., Portal B., Konstantinidis E., Eltom K., Libard S., Streubel-Gallasch L. et al. (2023) Astrocytic uptake of neuronal corpses promotes cell-to-cell spreading of tau pathology. Acta Neuropathol. Commun. 11, 97–97 10.1186/s40478-023-01589-8 37330529 PMC10276914

[30] Kim J., Yoo I.D., Lim J. and Moon J.S. (2024) Pathological phenotypes of astrocytes in Alzheimer’s disease. Exp. Mol. Med. 56, 95–99 10.1038/s12276-023-01148-0 38172603 PMC10834520

[31] Kapogiannis D. and Mattson M.P. (2011) Disrupted energy metabolism and neuronal circuit dysfunction in cognitive impairment and Alzheimer’s disease. Lancet Neurol. 10, 187–198 10.1016/S1474-4422(10)70277-5 21147038 PMC3026092

[32] Narasimhan S., Changolkar L., Riddle D.M., Kats A., Stieber A., Weitzman S.A. et al. (2019) Human tau pathology transmits glial tau aggregates in the absence of neuronal tau. J. Exp. Med. 217, e20190783–e20190783 10.1084/jem.20190783 PMC7041709 31826239

[33] Nagele R.G., Wegiel J., Venkataraman V., Imaki H., Wang K.C. and Wegiel J. (2004) Contribution of glial cells to the development of amyloid plaques in Alzheimer's disease. Neurobiol. Aging 25, 663–674 10.1016/j.neurobiolaging.2004.01.007 15172746

[34] Barbar L., Jain T., Zimmer M., Kruglikov I., Sadick J.S., Wang M. et al. (2020) CD49f Is a novel marker of functional and reactive human iPSC-derived astrocytes. Neuron 107, 436–453 10.1016/j.neuron.2020.05.014 32485136 PMC8274549

[35] Saroja S.R., Gorbachev K., TCW J., Goate A.M. and Pereira A.C. (2022) Astrocyte-secreted glypican-4 drives APOE4-dependent tau hyperphosphorylation. Proc. Natl. Acad. Sci. U.S.A. 199, 10.1073/pnas.2108870119 PMC9407658 35969759

[36] Singh D., Agrawal A., Singal C.M.S., Pandey H.S., Seth P. and Sharma S.K. (2020) Sinomenine inhibits amyloid beta-induced astrocyte activation and protects neurons against indirect toxicity. Mol. Brain 13, 10.1186/s13041-020-00569-6 32127013 PMC7055015

[37] Zhou L.T., Liu D., Kang H.C., Lu L., Huang H.Z., Ai W.Q. et al. (2023) Tau pathology epigenetically remodels the neuron-glial cross-talk in Alzheimer’s disease. Sci. Adv. 9, eaq7105 10.1126/sciadv.abq7105 PMC10121173 37083538

[38] Chen Y., Strickland M.R., Soranno A. and Holtzman D.M. (2021) Apolipoprotein E: structural insights and links to Alzheimer disease pathogenesis. Neuron 109, 205–221 10.1016/j.neuron.2020.10.008 33176118 PMC7931158

[39] Shi Y., Yamada K., Liddelow S.A., Smith S.T., Zhao L., Luo W. et al. (2017) ApoE4 markedly exacerbates tau-mediated neurodegeneration in a mouse model of tauopathy. Nature 549, 523–527 10.1038/nature24016 28959956 PMC5641217

[40] Wang C., Najm R., Xu Q., Jeong D.E., Walker D., Balestra M.E. et al. (2018) Gain of toxic apolipoprotein E4 effects in human iPSC-derived neurons is ameliorated by a small-molecule structure corrector. Nat. Med. 24, 647–657 10.1038/s41591-018-0004-z 29632371 PMC5948154

[41] Raman S., Brookhouser N. and Brafman D.A. (2020) Using human induced pluripotent stem cells (hiPSCs) to investigate the mechanisms by which Apolipoprotein E (APOE) contributes to Alzheimer’s disease (AD) risk. Neurobiol. Dis. 138, 104788 10.1016/j.nbd.2020.104788 32032733 PMC7098264

[42] Corona A.W., Kodoma N., Casali B.T. and Landreth G.E. (2016) ABCA1 is necessary for bexarotene-mediated clearance of soluble amyloid beta from the hippocampus of APP/PS1 mice. J. Neuroimmune Pharmacol. 11, 61–72 10.1007/s11481-015-9627-8 26175148 PMC6558963

[43] Lin Y.T., Seo J., Gao F., Feldman H.M., Wen H.L., Penney J. et al. (2018) APOE4 causes widespread molecular and cellular alterations associated with Alzheimer’s disease phenotypes in human iPSC-derived brain cell types. Neuron 98, 1141.e1147–1154.e1147 10.1016/j.neuron.2018.05.008 29861287 PMC6023751

[44] Pitas R.E., Boyles J.K., Lee S.H., Foss D. and Mahley R.W. (1987) Astrocytes synthesize apolipoprotein E and metabolize apolipoprotein E-containing lipoproteins. Biochim. Biophys. Acta Lipids Lipid Metab. 917, 148–161 10.1016/0005-2760(87)90295-5 3539206

[45] Sun Z., Kwon J.S., Ren Y., Chen S., Walker C.K., Lu X. et al. (2024) Modeling late-onset Alzheimer’s disease neuropathology via direct neuronal reprogramming. Science (New York, NY). 385,adl2992 10.1126/science.adl2992 PMC11787906 39088624

[46] Zhao J., Fu Y., Yamazaki Y., Ren Y., Davis M.D., Liu C.C. et al. (2020) APOE4 exacerbates synapse loss and neurodegeneration in Alzheimer’s disease patient iPSC-derived cerebral organoids. Nat. Commun. 11, 1–14 10.1038/s41467-020-19264-0 33139712 PMC7608683

[47] Zhao J., Davis M.D., Martens Y.A., Shinohara M., Graff-Radford N.R., Younkin S.G. et al. (2017) APOE ε4/ε4 diminishes neurotrophic function of human iPSC-derived astrocytes. Hum. Mol. Genet. 26, 2690–2690 10.1093/hmg/ddx155 28444230 PMC5886091

[48] Murakami R., Watanabe H., Hashimoto H., Kashiwagi-Hakozaki M., Hashimoto T., Karch C.M. et al. (2024) Inhibitory roles of apolipoprotein E christchurch astrocytes in curbing tau propagation using human pluripotent stem cell-derived models. J. Neurosci. 44 10.1523/JNEUROSCI.1709-23.2024 38649269 PMC11170944

[49] Mothes T., Konstantinidis E., Eltom K., Dakhel A., Rostami J. and Erlandsson A. (2024) Tau processing and tau-mediated inflammation differ in human APOEε2 and APOEε4 astrocytes. iScience 27, 111163–111163 10.1016/j.isci.2024.111163 39524360 PMC11549983

[50] Gomes C., Huang K.C., Harkin J., Baker A., Hughes J.M., Pan Y. et al. (2024) Induction of astrocyte reactivity promotes neurodegeneration in human pluripotent stem cell models. Stem Cell Rep. 19, 1122–1136 10.1016/j.stemcr.2024.07.002 39094561 PMC11368677

[51] Sun G.G., Wang C., Mazzarino R.C., Perez-Corredor P.A., Davtyan H., Blurton-Jones M. et al. (2024) Microglial APOE3 Christchurch protects neurons from Tau pathology in a human iPSC-based model of Alzheimer’s disease. Cell Rep. 43, 114982–114982, 10.1016/j.celrep.2024.114982 39612244 PMC11753789

[52] Fleeman R.M. and Proctor E.A. (2021) Astrocytic propagation of tau in the context of Alzheimer’s disease. Front. Cell. Neurosci. 15, 645233–645233 10.3389/fncel.2021.645233 33815065 PMC8010320

[53] Beretta C., Svensson E., Dakhel A., Zyśk M., Hanrieder J., Sehlin D. et al. (2024) Amyloid-β deposits in human astrocytes contain truncated and highly resistant proteoforms. Mol. Cell. Neurosci. 128, 103916–103916 10.1016/j.mcn.2024.103916 38244652

[54] Eltom K., Mothes T., Libard S., Ingelsson M. and Erlandsson A. (2024) Astrocytic accumulation of tau fibrils isolated from Alzheimer’s disease brains induces inflammation, cell-to-cell propagation and neuronal impairment. Acta Neuropathol. Commun. 12, 34–34 10.1186/s40478-024-01745-8 38409026 PMC10898102

[55] Rickner H.D., Jiang L., Hong R., O'Neill N.K., Mojica C.A., Snyder B.J. et al. (2022) Single cell transcriptomic profiling of a neuron-astrocyte assembloid tauopathy model. Nat. Commun. 13, 6275 10.1038/s41467-022-34005-1 36271092 PMC9587045

[56] Liao M.-C., Muratore C.R., Gierahn T.M., Sullivan S.E., Srikanth P., De Jager P.L. et al. (2016) Single-cell detection of secreted Aβ and sAPPα from human IPSC-derived neurons and astrocytes. Neurobiol. Dis. 36, 1730–46 26843653 10.1523/JNEUROSCI.2735-15.2016 PMC4737781

[57] Mulica P., Grünewald A. and Pereira S.L. (2021) Astrocyte-neuron metabolic crosstalk in neurodegeneration: a mitochondrial perspective. Front. Endocrinol. 12, 668517–668517 10.3389/fendo.2021.668517 PMC8138625 34025580

[58] MacMullen C., Sharma N. and Davis R.L. (2025) Mitochondrial dynamics and bioenergetics in Alzheimer’s induced pluripotent stem cell-derived neurons. Brain 148, 1405–1420 10.1093/brain/awae364 39513728 PMC12168126

[59] Konttinen H., Gureviciene I., Oksanen M., Grubman A., Loppi S., Huuskonen M.T. et al. (2018) PPARβ/δ-agonist GW0742 ameliorates dysfunction in fatty acid oxidation in PSEN1ΔE9 astrocytes. Glia 67, 146–146 10.1002/glia.23534 30453390 PMC7526864

[60] Oksanen M., Petersen A.J., Naumenko N., Puttonen K., Lehtonen Š., Gubert Olivé M. et al. (2017) PSEN1 mutant iPSC-derived model reveals severe astrocyte pathology in Alzheimer’s disease. Stem Cell Rep. 9, 1885–1897 10.1016/j.stemcr.2017.10.016 29153989 PMC5785689

[61] Flannagan K., Stopperan J.A., Hauger B.M., Troutwine B.R., Lysaker C.R., Strope T.A. et al. (2023) Cell type and sex specific mitochondrial phenotypes in iPSC derived models of Alzheimer’s disease. Front. Mol. Neurosci. 16, 1201015–1201015 10.3389/fnmol.2023.1201015 37614699 PMC10442646

[62] Coronel R., García-Moreno E., Siendones E., Barrero M.J., Martínez-Delgado B., Santos-Ocaña C. et al. (2024) Brain organoid as a model to study the role of mitochondria in neurodevelopmental disorders: achievements and weaknesses. Front. Cell. Neurosci. 18, 1403734–1403734 10.3389/fncel.2024.1403734 38978706 PMC11228165

[63] Lampinen R., Belaya I., Saveleva L., Liddell J.R., Rait D., Huuskonen M.T. et al. (2022) Neuron-astrocyte transmitophagy is altered in Alzheimer’s disease. Neurobiol. Dis. 170 10.1016/j.nbd.2022.105753 35569719

[64] Wyss-Coray T., Lin C., Yan F., Yu G.Q., Rohde M., McConlogue L. et al. (2001) TGF-β1 promotes microglial amyloid-β clearance and reduces plaque burden in transgenic mice. Nat. Med. 7, 612–618 10.1038/87945 11329064

[65] Cai Y., Liu J., Wang B., Sun M. and Yang H. (2022) Microglia in the neuroinflammatory pathogenesis of Alzheimer’s disease and related therapeutic targets. Front. Immunol. 13, 856376–856376 10.3389/fimmu.2022.856376 35558075 PMC9086828

[66] Keren-Shaul H., Spinrad A., Weiner A., Matcovitch-Natan O., Dvir-Szternfeld R., Ulland T.K. et al. (2017) A unique microglia type associated with restricting development of Alzheimer’s disease. Cell 169, 1276.e1217–1290.e1217 10.1016/j.cell.2017.05.018 28602351

[67] Dejanovic B., Wu T., Tsai M.C., Graykowski D., Gandham V.D., Rose C.M. et al. (2022) Complement C1q-dependent excitatory and inhibitory synapse elimination by astrocytes and microglia in Alzheimer’s disease mouse models. Nat. Aging 2, 837–850 10.1038/s43587-022-00281-1 37118504 PMC10154216

[68] Hong S., Beja-Glasser V.F., Nfonoyim B.M., Frouin A., Li S., Ramakrishnan S. et al. (2016) Complement and microglia mediate early synapse loss in Alzheimer mouse models. Science (New York, NY) 352, 712–712 10.1126/science.aad8373 PMC5094372 27033548

[69] Nizami S., Hall-Roberts H., Warrier S., Cowley S.A. and Di Daniel E. (2019) Microglial inflammation and phagocytosis in Alzheimer’s disease: potential therapeutic targets. Br. J. Pharmacol. 176, 3515–3515 10.1111/bph.14618 30740661 PMC6715590

[70] Abud E.M., Ramirez R.N., Martinez E.S., Healy L.M., Nguyen C.H.H., Newman S.A. et al. (2017) iPSC-derived human microglia-like cells to study neurological diseases. Neuron 94, 278.e279–293.e279 10.1016/j.neuron.2017.03.042 28426964 PMC5482419

[71] Dolan M.J., Therrien M., Jereb S., Kamath T., Gazestani V., Atkeson T. et al. (2023) Exposure of iPSC-derived human microglia to brain substrates enables the generation and manipulation of diverse transcriptional states *in vitro*. Nat. Immunol. 24, 1382–1382 10.1038/s41590-023-01558-2 37500887 PMC10382323

[72] Azizi G., Khannazer N. and Mirshafiey A. (2014) The potential role of chemokines in Alzheimer’s disease pathogenesis. Am. J. Alzheimers Dis. Other Demen. 29, 415–415 10.1177/1533317513518651 24408754 PMC10852600

[73] Corrêa J.D., Starling D., Teixeira A.L., Caramelli P. and Silva T.A. (2011) Chemokines in CSF of Alzheimer’s disease patients. Arq. Neuropsiquiatr. 69, 455–459 10.1590/S0004-282X2011000400009 21755121

[74] Guttikonda S.R., Sikkema L., Tchieu J., Saurat N., Walsh R.M., Harschnitz O. et al. (2021) Fully defined human pluripotent stem cell-derived microglia and tri-culture system model C3 production in Alzheimer’s disease. Nat. Neurosci. 24, 343–343 10.1038/s41593-020-00796-z 33558694 PMC8382543

[75] Park J., Wetzel I., Marriott I., Dréau D., D'Avanzo C., Kim D.Y. et al. (2018) A 3D human triculture system modeling neurodegeneration and neuroinflammation in Alzheimer’s disease. Nat. Neurosci. 21, 941–951 10.1038/s41593-018-0175-4 29950669 PMC6800152

[76] Victor M.B., Leary N., Luna X., Meharena H.S., Scannail A.N., Bozzelli P.L. et al. (2022) Lipid accumulation induced by APOE4 impairs microglial surveillance of neuronal-network activity. Cell Stem Cell 29, 1197.e1198–1212.e1198 10.1016/j.stem.2022.07.005 35931030 PMC9623845

[77] Mordelt A. and de Witte L.D. (2023) Microglia-mediated synaptic pruning as a key deficit in neurodevelopmental disorders: hype or hope? Curr. Opin. Neurobiol. 79, 102674–102674 10.1016/j.conb.2022.102674 36657237

[78] Johnson S.A., Pasinetti G.M. and Finch C.E. (1994) Expression of complement C1qB and C4 mRNAs during rat brain development. Brain Res. Dev. Brain Res. 80, 163–174 10.1016/0165-3806(94)90101-5 7955342

[79] Reichwald J., Danner S., Wiederhold K.H. and Staufenbiel M. (2009) Expression of complement system components during aging and amyloid deposition in APP transgenic mice. J. Neuroinflammation. 6, 35 10.1186/1742-2094-6-35 19917141 PMC2784442

[80] Schafer D.P., Lehrman E.K., Kautzman A.G., Koyama R., Mardinly A.R., Yamasaki R. et al. (2012) Microglia sculpt postnatal neural circuits in an activity and complement-dependent manner. Neuron 74, 691–705 10.1016/j.neuron.2012.03.026 22632727 PMC3528177

[81] Penney J., Ralvenius W.T., Loon A., Cerit O., Dileep V., Milo B. et al. (2024) iPSC-derived microglia carrying the TREM2 R47H/+ mutation are proinflammatory and promote synapse loss. Glia 72, 452–469 10.1002/glia.24485 37969043 PMC10904109

[82] Lish A.M., Ashour N., Pearse R.V., Galle P.C., Orme G.A., Heuer S.E. et al. (2025) Astrocyte induction of disease-associated microglia is suppressed by acute exposure to fAD neurons in human iPSC triple cultures. Cell Rep. 44 10.1016/j.celrep.2025.115777 40471789 PMC12282607

[83] Lish A.M., Grogan E.F.L., Benoit C.R., Pearse R.V., Heuer S.E., Luquez T. et al. (2025) CLU alleviates Alzheimer’s disease-relevant processes by modulating astrocyte reactivity and microglia-dependent synaptic density. Neuron 113, 1925.e1911–1946.e1911 40311610 10.1016/j.neuron.2025.03.034 PMC12181066

[84] Su D., Cui Y., He C., Yin P., Bai R., Zhu J. et al. (2025) Projections for prevalence of Parkinson’s disease and its driving factors in 195 countries and territories to 2050: modelling study of Global Burden of Disease Study 2021. BMJ 388, e080952–e080952 10.1136/bmj-2024-080952 40044233 PMC11881235

[85] Ray Dorsey E., Elbaz A., Nichols E., Abd-Allah F., Abdelalim A., Adsuar J.C. et al. (2018) Global, regional, and national burden of Parkinson’s disease, 1990-2016: a systematic analysis for the Global Burden of Disease Study 2016. Lancet Neurol. 17, 939–953 30287051 10.1016/S1474-4422(18)30295-3 PMC6191528

[86] Kalia L.V. and Lang A.E. (2015) Parkinson’s disease. Lancet North Am. Ed. 386, 896–912 10.1016/S0140-6736(14)61393-3 25904081

[87] Morris H.R., Spillantini M.G., Sue C.M. and Williams-Gray C.H. (2024) The pathogenesis of Parkinson’s disease. Lancet North Am. Ed. 403, 293–304 10.1016/S0140-6736(23)01478-2 38245249

[88] Braak H., Del Tredici K., Rüb U., De Vos R.A.I., Jansen Steur E.N.H. and Braak E. (2003) Staging of brain pathology related to sporadic Parkinson’s disease. Neurobiol. Aging 24, 197–211 10.1016/S0197-4580(02)00065-9 12498954

[89] Kam T.I., Hinkle J.T., Dawson T.M. and Dawson V.L. (2020) Microglia and astrocyte dysfunction in Parkinson’s disease. Neurobiol. Dis. 144, 105028–105028 10.1016/j.nbd.2020.105028 32736085 PMC7484088

[90] Pierce S. and Coetzee G.A. (2017) Parkinson’s disease-associated genetic variation is linked to quantitative expression of inflammatory genes. PloS One 12, ​​e0175882 28407015 10.1371/journal.pone.0175882 PMC5391096

[91] Nalls M.A., Pankratz N., Lill C.M., Do C.B., Hernandez D.G., Saad M. et al. (2014) Large-scale meta-analysis of genome-wide association data identifies six new risk loci for Parkinson’s disease. Nat. Genet. 46, 989–989 10.1038/ng.3043 25064009 PMC4146673

[92] Nalls M.A., Blauwendraat C., Vallerga C.L., Heilbron K., Bandres-Ciga S., Chang D. et al. (2019) Identification of novel risk loci, causal insights, and heritable risk for Parkinson’s disease: a meta-analysis of genome-wide association studies. Lancet Neurol. 18, 1091–1102 10.1016/S1474-4422(19)30320-5 31701892 PMC8422160

[93] Wang N., Xiao X., Chen Z., Xu K., Cao X., Kou D. et al. (2025) Glial cell crosstalk in Parkinson’s disease: mechanisms, implications, and therapeutic strategies. Fundamental Res. 5, 2960–2974 10.1016/j.fmre.2024.12.023 PMC12744648 41466978

[94] Booth H.D.E., Hirst W.D. and Wade-Martins R. (2017) The role of astrocyte dysfunction in Parkinson’s disease pathogenesis. Trends Neurosci. 40, 358–358 10.1016/j.tins.2017.04.001 28527591 PMC5462417

[95] Chang D., Nalls M.A., Halgrímsdóttir I.B., Hunkapiller J., Brug M.v.d., Cai F. et al. (2017) A meta-analysis of genome-wide association studies identifies 17 new Parkinson’s disease risk loci. Nat. Genet. 49, 1511–1516 10.1038/ng.3955 28892059 PMC5812477

[96] Real R., Martinez-Carrasco A., Reynolds R.H., Lawton M.A., Tan M.M.X., Shoai M. et al. (2023) Association between the LRP1B and APOE loci and the development of Parkinson’s disease dementia. Brain 146, 1873–1887 10.1093/brain/awac414 36348503 PMC10151192

[97] Zhang Y., Sloan S.A., Clarke L.E., Caneda C., Plaza C.A., Blumenthal P.D. et al. (2016) Purification and characterization of progenitor and mature human astrocytes reveals transcriptional and functional differences with mouse. Neuron 89, 37–53 10.1016/j.neuron.2015.11.013 26687838 PMC4707064

[98] Yvanka de Soysa T., Thrrien M., Walker A.C. and Stevens B. (2022) Redefining microglia states: lessons and limits of human and mouse models to study microglia states in neurodegenerative diseases. Semin. Immunol. 60, 101651–101651 10.1016/j.smim.2022.101651 36155944

[99] Miyazaki I. and Asanuma M. (2020) Neuron-astrocyte interactions in Parkinson’s disease. Cells 9, 1–28 10.3390/cells9122623 PMC7762285 33297340

[100] Du F., Yu Q., Chen A., Chen D. and ShiDu Yan S. (2018) Astrocytes attenuate mitochondrial dysfunctions in human dopaminergic neurons derived from iPSC. Stem Cell Rep. 10, 366–366 10.1016/j.stemcr.2017.12.021 29396183 PMC5830955

[101] Yang F., Liu Y., Tu J., Wan J., Zhang J., Wu B. et al. (2014) Activated astrocytes enhance the dopaminergic differentiation of stem cells and promote brain repair through bFGF. Nat. Commun. 5, 5627 10.1038/ncomms6627 25517983 PMC4284631

[102] Srinivasan E., Chandrasekhar G., Chandrasekar P., Anbarasu K., Vickram A.S., Karunakaran R. et al. (2021) Alpha-synuclein aggregation in Parkinson’s disease. Front. Med. 8, 736978–736978 10.3389/fmed.2021.736978 PMC8558257 34733860

[103] Tsunemi T., Ishiguro Y., Yoroisaka A., Valdez C., Miyamoto K., Ishikawa K. et al. (2020) Astrocytes protect human dopaminergic neurons from α-synuclein accumulation and propagation. J. Neurosci. 40, 8618–8628 10.1523/JNEUROSCI.0954-20.2020 33046546 PMC7643299

[104] Aflaki E., Stubblefield B.K., McGlinchey R.P., McMahon B., Ory D.S. and Sidransky E. (2019) A characterization of Gaucher iPS-derived astrocytes: potential implications for Parkinson disease. Neurobiol. Dis. 134, 104647–104647 10.1016/j.nbd.2019.104647 31669751 PMC6980699

[105] Weiss F., Hughes L., Fu Y., Bardy C., Halliday G.M. and Dzamko N. (2024) Astrocytes contribute to toll-like receptor 2-mediated neurodegeneration and alpha-synuclein pathology in a human midbrain Parkinson’s model. Transl. Neurodegener. 13, 62–62 10.1186/s40035-024-00448-3 39681872 PMC11648304

[106] di Domenico A., Carola G., Calatayud C., Pons-Espinal M., Muñoz J.P., Richaud-Patin Y. et al. (2019) Patient-specific iPSC-derived astrocytes contribute to non-cell-autonomous neurodegeneration in Parkinson’s disease. Stem Cell Rep. 12, 213–213 10.1016/j.stemcr.2018.12.011 30639209 PMC6372974

[107] Jacquet A.d.R., Tancredi J.L., Lemire A.L., Desantis M.C., Li W.P. and O'shea E.K. (2021) The LRRK2 G2019S mutation alters astrocyte-to-neuron communication via extracellular vesicles and induces neuron atrophy in a human iPSC-derived model of Parkinson’s disease. eLife. 10, e73062 34590578 10.7554/eLife.73062 PMC8514240

[108] Cheng X.Y., Biswas S., Li J., Mao C.J., Chechneva O., Chen J. et al. (2020) Human iPSCs derived astrocytes rescue rotenone-induced mitochondrial dysfunction and dopaminergic neurodegeneration* in vitro* by donating functional mitochondria. Transl. Neurodegener. 9, 13 10.1186/s40035-020-00190-6 32345341 PMC7325238

[109] Russ K., Teku G., Bousset L., Redeker V., Piel S., Savchenko E. et al. (2021) TNF-α and α-synuclein fibrils differently regulate human astrocyte immune reactivity and impair mitochondrial respiration. Cell Rep. 34, 108895 10.1016/j.celrep.2021.108895 33761362

[110] Schmidt S.I., Bogetofte H., Ritter L., Agergaard J.B., Hammerich D., Kabiljagic A.A. et al. (2021) Microglia-secreted factors enhance dopaminergic differentiation of tissue- and iPSC-derived human neural stem cells. Stem Cell Rep. 16, 281–281 10.1016/j.stemcr.2020.12.011 33482100 PMC7878834

[111] Zhang X., Yu H. and Feng J. (2024) Emerging role of microglia in inter-cellular transmission of α-synuclein in Parkinson’s disease. Front. Aging Neurosci. 16, 1411104–1411104 10.3389/fnagi.2024.1411104 39444806 PMC11496080

[112] Scheiblich H., Dansokho C., Mercan D., Schmidt S.V., Bousset L., Wischhof L. et al. (2021) Microglia jointly degrade fibrillar alpha-synuclein cargo by distribution through tunneling nanotubes. Cell 184, 5089–5089 10.1016/j.cell.2021.09.007 34555357 PMC8527836

[113] Raposo G. and Stoorvogel W. (2013) Extracellular vesicles: exosomes, microvesicles, and friends. J. Cell Biol. 200, 373–373 10.1083/jcb.201211138 23420871 PMC3575529

[114] Guo M., Wang J., Zhao Y., Feng Y., Han S., Dong Q. et al. (2020) Microglial exosomes facilitate α‐synuclein transmission in Parkinson’s disease. Brain 143, 1476–1497 10.1093/brain/awaa090 32355963 PMC7241957

[115] Tu H.Y., Yuan B., Hou X., Zhang X., Pei C., Ma Y. et al. (2021) α-Synuclein suppresses microglial autophagy and promotes neurodegeneration in a mouse model of Parkinson’s disease. Aging Cell. 20, e13522 10.1111/acel.13522 34811872 PMC8672776

[116] Tang Q., Gao P., Arzberger T., Höllerhage M., Hermes J., Höglinger G. et al. (2021) Alpha‐synuclein defects autophagy by impairing SNAP29‐mediated autophagosome‐lysosome fusion. Cell Death Dis. 12, 854 10.1038/s41419-021-04138-0 34535638 PMC8448865

[117] Xia Y., Zhang G., Han C., Ma K., Guo X., Wan F. et al. (2019) Microglia as modulators of exosomal alpha-synuclein transmission. Cell Death Dis. 10, 174 10.1038/s41419-019-1404-9 30787269 PMC6382842

[118] Abe T., Kuwahara T., Suenaga S., Sakurai M., Takatori S. and Iwatsubo T. (2024) Lysosomal stress drives the release of pathogenic α-synuclein from macrophage lineage cells via the LRRK2-Rab10 pathway. iScience 27, 108893 10.1016/j.isci.2024.108893 38313055 PMC10835446

[119] Gerrits E., Heng Y., Boddeke E.W.G.M. and Eggen B.J.L. (2019) Transcriptional profiling of microglia; current state of the art and future perspectives. Glia 68, 740–740 10.1002/glia.23767 31846124 PMC7064956

[120] Hasselmann J. and Blurton-Jones M. (2020) Human iPSC-derived microglia: a growing toolset to study the brain’s innate immune cells. Glia 68, 721–721 10.1002/glia.23781 31926038 PMC7813153

[121] Trainor A.R., MacDonald D.S. and Penney J. (2024) Microglia: roles and genetic risk in Parkinson’s disease. Front. Neurosci. 18, 1506358–1506358 10.3389/fnins.2024.1506358 39554849 PMC11564156

[122] Panagiotakopoulou V., Ivanyuk D., De Cicco S., Haq W., Arsić A., Yu C. et al. (2020) Interferon-γ signaling synergizes with LRRK2 in neurons and microglia derived from human induced pluripotent stem cells. Nat. Commun. 11, 5163–5163 10.1038/s41467-020-18755-4 33057020 PMC7560616

[123] Trudler D., Nazor K.L., Eisele Y.S., Grabauskas T., Dolatabadi N., Parker J. et al. (2021) Soluble α-synuclein-antibody complexes activate the NLRP3 inflammasome in hiPSC-derived microglia. Proc. Natl. Acad. Sci. U.S.A. 118, e2025847118–e2025847118 10.1073/pnas.2025847118 33833060 PMC8054017

[124] Imamura K., Hishikawa N., Sawada M., Nagatsu T., Yoshida M. and Hashizume Y. (2003) Distribution of major histocompatibility complex class II-positive microglia and cytokine profile of Parkinson’s disease brains. Acta Neuropathol. (Berl) 106, 518–526 10.1007/s00401-003-0766-2 14513261

[125] Blasco-Agell L., Pons-Espinal M., Testa V., Roch G., Montero-Muñoz J., Fernandez-Carasa I. et al. (2025) LRRK2-mutant microglia and neuromelanin synergize to drive dopaminergic neurodegeneration in an iPSC-based Parkinson’s disease model. Commun. Biol. 8, 10.1038/s42003-025-08544-4 40796643 PMC12344146

[126] Smajic S., Prada-Medina C.A., Landoulsi Z., Ghelfi J., Delcambre S., Dietrich C. et al. (2022) Single-cell sequencing of human midbrain reveals glial activation and a Parkinson-specific neuronal state. Brain 145, 964–978 10.1093/brain/awab446 34919646 PMC9050543

[127] Zhang Y., Wu K.M., Yang L., Dong Q. and Yu J.T. (2022) Tauopathies: new perspectives and challenges. Mol. Neurodegener. 17, 1–29 10.1186/s13024-022-00533-z 35392986 PMC8991707

[128] Lee V.M.Y., Goedert M. and Trojanowski J.Q. (2001) Neurodegenerative tauopathies. Ann. Rev. Neurosci. 56, 43–43 10.1146/annurev.neuro.24.1.1121 11520930

[129] Guven G., Lohmann E., Bras J., Gibbs J.R., Gurvit H., Bilgic B. et al. (2016) Mutation frequency of the major frontotemporal dementia genes, MAPT, GRN and C9ORF72 in a Turkish cohort of dementia patients. PloS One 11, e0162592–e0162592 10.1371/journal.pone.0162592 27632209 PMC5025192

[130] Takada L.T. (2015) The genetics of monogenic frontotemporal dementia. Dementia Neuropsychol. 9, 219–219 10.1590/1980-57642015dn93000003 29213965 PMC5619362

[131] Chung D.e.C., Roemer S., Petrucelli L. and Dickson D.W. (2021) Cellular and pathological heterogeneity of primary tauopathies. Mol. Neurodegener. 16, 1–20 10.1186/s13024-021-00476-x 34425874 PMC8381569

[132] Armstrong M.J., Litvan I., Lang A.E., Bak T.H., Bhatia K.P., Borroni B. et al. (2013) Criteria for the diagnosis of corticobasal degeneration. Neurology 80, 496–503 10.1212/WNL.0b013e31827f0fd1 23359374 PMC3590050

[133] Kovacs G.G., Lukic M.J., Irwin D.J., Arzberger T., Respondek G., Lee E.B. et al. (2020) Distribution patterns of tau pathology in progressive supranuclear palsy. Acta Neuropathol. (Berl) 140, 99–119 10.1007/s00401-020-02158-2 32383020 PMC7360645

[134] Supakul S., Murakami R., Oyama C., Shindo T., Hatakeyama Y., Itsuno M. et al. (2024) Mutual interaction of neurons and astrocytes derived from iPSCs with APP V717L mutation developed the astrocytic phenotypes of Alzheimer’s disease. Inflamm. Regen. 44, 1–21 10.1186/s41232-023-00310-5 38419091 PMC10900748

[135] Sloan S.A., Darmanis S., Huber N., Khan T.A., Birey F., Caneda C. et al. (2017) Human astrocyte maturation captured in 3D cerebral cortical spheroids derived from pluripotent stem cells. Neuron 95, 779.e776–790.e776 10.1016/j.neuron.2017.07.035 28817799 PMC5890820

[136] Voulgaris D., Nikolakopoulou P. and Herland A. (2022) Generation of human iPSC-derived astrocytes with a mature star-shaped phenotype for CNS modeling. Stem Cell Rev. Rep. 18, 2494–2512 10.1007/s12015-022-10376-2 35488987 PMC9489586

[137] Tcw J., Wang M., Pimenova A.A., Bowles K.R., Hartley B.J., Lacin E. et al. (2017) An efficient platform for astrocyte differentiation from human induced pluripotent stem cells. Stem Cell Rep. 9, 600–614 10.1016/j.stemcr.2017.06.018 28757165 PMC5550034

[138] Hedegaard A., Monzón-Sandoval J., Newey S.E., Whiteley E.S., Webber C. and Akerman C.J. (2020) Pro-maturational effects of human iPSC-derived cortical astrocytes upon iPSC-derived cortical neurons. Stem Cell Rep. 15, 38–51 10.1016/j.stemcr.2020.05.003 32502466 PMC7363746

[139] Sidoryk-Wegrzynowicz M., Gerber Y.N., Ries M., Sastre M., Tolkovsky A.M. and Spillantini M.G. (2017) Astrocytes in mouse models of tauopathies acquire early deficits and lose neurosupportive functions. Acta Neuropathol. Commun. 5, 89–89 10.1016/S0140-6736(14)61393-3 29187256 PMC6389177

[140] Hampton D.W., Webber D.J., Bilican B., Goedert M., Spillantini M.G. and Chandran S. (2010) Cell-mediated neuroprotection in a mouse model of human tauopathy. J. Neurosci. 30, 9973–9973 10.1523/JNEUROSCI.0834-10.2010 20668182 PMC6633376

[141] Ezerskiy L.A., Schoch K.M., Sato C., Beltcheva M., Horie K., Rigo F. et al. (2022) Astrocytic 4R tau expression drives astrocyte reactivity and dysfunction. JCI Insight 7​​​​​, e152012 10.1172/jci.insight.152012 34874917 PMC8765054

[142] Batenburg K.L., Kasri N.N., Heine V.M. and Scheper W. (2022) Intraneuronal tau aggregation induces the integrated stress response in astrocytes. J. Mol. Cell Biol. 14, mjac071 10.1093/jmcb/mjac071 PMC10080549 36520068

[143] Vogels T., Murgoci A.N. and Hromádka T. (2019) Intersection of pathological tau and microglia at the synapse. Acta Neuropathol. Commun. 7, 1–25 10.1186/s40478-019-0754-y PMC6612163 31277708

[144] Laurent C., Buée L. and Blum D. (2018) Tau and neuroinflammation: what impact for Alzheimer’s disease and tauopathies? Biomed. J. 41, 21–21 10.1016/j.bj.2018.01.003 29673549 PMC6138617

[145] Palleis C., Sauerbeck J., Beyer L., Harris S., Schmitt J., Morenas-Rodriguez E. et al. (2021) *In vivo* assessment of neuroinflammation in 4-repeat tauopathies. Mov. Disord. 36, 883–894 10.1002/mds.28395 33245166

[146] Asai H., Ikezu S., Tsunoda S., Medalla M., Luebke J., Haydar T. et al. (2015) Depletion of microglia and inhibition of exosome synthesis halt tau propagation. Nat. Neurosci. 18, 1584–1593 10.1038/nn.4132 26436904 PMC4694577

[147] Brelstaff J., Tolkovsky A.M., Ghetti B., Goedert M. and Spillantini M.G. (2018) Living neurons with tau filaments aberrantly expose phosphatidylserine and are phagocytosed by microglia. Cell Rep. 24, 1939.e1934–1948.e1934 10.1016/j.celrep.2018.07.072 30134156 PMC6161320

[148] Iyer A.K., Vermunt L., Mirfakhar F.S., Minaya M., Acquarone M., Koppisetti R.K. et al. (2025) Cell autonomous microglia defects in a stem cell model of frontotemporal dementia tau. Mol. Psychiatry 1–16 10.1038/s41380-025-03073-2 PMC12436154 40527900

[149] Simons M. and Nave K.A. (2015) Oligodendrocytes: myelination and axonal support. Cold Spring Harb. Perspect. Biol. 8, a020479 10.1101/cshperspect.a020479 26101081 PMC4691794

[150] Shim G., Romero-Morales A.I., Sripathy S.R. and Maher B.J. (2024) Utilizing hiPSC-derived oligodendrocytes to study myelin pathophysiology in neuropsychiatric and neurodegenerative disorders. Front. Cell. Neurosci. 17, 1322813–1322813 10.3389/fncel.2023.1322813 38273973 PMC10808804

[151] Dehestani M., Kozareva V., Blauwendraat C., Fraenkel E., Gasser T. and Bansal V. (2024) Transcriptomic changes in oligodendrocytes and precursor cells associate with clinical outcomes of Parkinson’s disease. Mol. Brain 17, 1–10 10.1186/s13041-024-01128-z 39138468 PMC11323592

[152] Bryois J., Skene N.G., Hansen T.F., Kogelman L.J.A., Watson H.J., Liu Z. et al. (2020) Genetic identification of cell types underlying brain complex traits yields insights into the etiology of Parkinson’s disease. Nat. Genet. 52, 482–493 10.1038/s41588-020-0610-9 32341526 PMC7930801

[153] Salazar Campos J.M., Burbulla L.F. and Jäkel S. (2025) Are oligodendrocytes bystanders or drivers of Parkinson’s disease pathology? PLoS Biol. 23, e3002977–e3002977 10.1371/journal.pbio.3002977 39777410 PMC11709285

[154] Wang Q., Antone J., Alsop E., Reiman R., Funk C., Bendl J. et al. (2024) Single cell transcriptomes and multiscale networks from persons with and without Alzheimer’s disease. Nat. Commun. 15, 1–16 38987616 10.1038/s41467-024-49790-0 PMC11237088

[155] Adams L., Song M.K., Yuen S., Tanaka Y. and Kim Y.S. (2024) A single-nuclei paired multiomic analysis of the human midbrain reveals age- and Parkinson’s disease-associated glial changes. Nat. Aging 4, 364–378 10.1038/s43587-024-00583-6 38491288 PMC11361719

[156] Bae E.J., Pérez-Acuña D., Rhee K.H. and Lee S.J. (2023) Changes in oligodendroglial subpopulations in Parkinson’s disease. Mol. Brain 16, 1–4 10.1186/s13041-023-01055-5 37710343 PMC10500805

[157] Ramirez A.M., Nasciben L.B., Moura S., Coombs L., Rajabli F., DeRosa B.A. et al. (2025) Ancestral genomic functional differences in oligodendroglia: implications for Alzheimer’s disease. Alzheimers Dement. 21, e70593 40937943 10.1002/alz.70593 PMC12426912

[158] Cai Y., Pinheiro-de-Sousa I., Slobodyanyuk M., Chen F., Huynh T., Kanyo J. et al. (2025) Myelin-axon interface vulnerability in Alzheimer’s disease revealed by subcellular proteomics and imaging of human and mouse brain. Nat. Neurosci. 28, 1418–1435 10.1038/s41593-025-01973-8 40514588 PMC12395420

[159] Albert K., Niskanen J., Kälvälä S. and Lehtonen Š. (2021) Utilising induced pluripotent stem cells in neurodegenerative disease research: focus on glia. Int. J. Mol. Sci. 22, 4334–4334 10.3390/ijms22094334 33919317 PMC8122303

[160] Braak H., Tredici K.D., Gai W.P. and Braak E. (2000) Alpha-synuclein is not a requisite component of synaptic boutons in the adult human central nervous system. J. Chem. Neuroanat. 20, 245–252 10.1016/S0891-0618(00)00101-0 11207422

[161] Farrell K., Humphrey J., Chang T., Zhao Y., Leung Y.Y., Kuksa P.P. et al. (2024) Genetic, transcriptomic, histological, and biochemical analysis of progressive supranuclear palsy implicates glial activation and novel risk genes. Nat. Commun. 15, 1–17 39251599 10.1038/s41467-024-52025-x PMC11385559

[162] Miedema S.S.M., Mol M.O., Koopmans F.T.W., Hondius D.C., van Nierop P., Menden K. et al. (2022) Distinct cell type-specific protein signatures in GRN and MAPT genetic subtypes of frontotemporal dementia. Acta Neuropathol. Commun. 10, 100–100 10.1186/s40478-022-01387-8 35799292 PMC9261008

[163] Viney T.J., Sarkany B., Ozdemir A.T., Hartwich K., Schweimer J., Bannerman D. et al. (2022) Spread of pathological human Tau from neurons to oligodendrocytes and loss of high-firing pyramidal neurons in aging mice. Cell Rep. 41, 111646–111646 10.1016/j.celrep.2022.111646 36384116 PMC9681663

[164] Seiberlich V., Bauer N.G., Schwarz L., Ffrench-Constant C., Goldbaum O. and Richter-Landsberg C. (2015) Downregulation of the microtubule associated protein Tau impairs process outgrowth and myelin basic protein mRNA transport in oligodendrocytes. Glia 63, 1621–1635 10.1002/glia.22832 25847153

[165] Chie S.E., Szentpetery Z., Generali M., Kuhlmann T., Natalucci G. and Miletta M.C. (2025) Human iPSC-derived neuron and oligodendrocyte co-culture as a small-molecule screening assay for myelination. Bio Protoc. 15, e5227–e5227 10.21769/BioProtoc.5227 40364981 PMC12067306

[166] Ehrlich M., Mozafari S., Glatza M., Starost L., Velychko S., Hallmann A.L. et al. (2017) Rapid and efficient generation of oligodendrocytes from human induced pluripotent stem cells using transcription factors. Proc. Natl. Acad. Sci. U.S.A. 114, E2243–E2252, 10.1073/pnas.1614412114 28246330 PMC5358375

[167] García-León J.A., Kumar M., Boon R., Chau D., One J., Wolfs E. et al. (2018) SOX10 single transcription factor-based fast and efficient generation of oligodendrocytes from human pluripotent stem cells. Stem Cell Rep. 10, 655–672 10.1016/j.stemcr.2017.12.014 29337119 PMC5830935

[168] von der Bey M., De Cicco S., Zach S., Hengerer B. and Ercan-Herbst E. (2023) Three-dimensional co-culture platform of human induced pluripotent stem cell-derived oligodendrocyte lineage cells and neurons for studying myelination. STAR Protoc. 4, 102164 36933222 10.1016/j.xpro.2023.102164 PMC10034497

[169] Assetta B., Tang C., Bian J., O'Rourke R., Connolly K., Brickler T. et al. (2020) Generation of human neurons and oligodendrocytes from pluripotent stem cells for modeling neuron-oligodendrocyte interactions. J. Vis. Exp. 2020, 1–15 10.3791/61778 PMC7739515 33226027

[170] Dooves S., Nadadhur A.G., Gasparotto L. and Heine V.M. (2019) Co-culture of human stem cell derived neurons and oligodendrocyte progenitor cells. Bio Protoc. 9, e3350–e3350 10.21769/BioProtoc.3350 33654852 PMC7854112

[171] Shaker M.R., Pietrogrande G., Martin S., Lee J.H., Sun W. and Wolvetang E.J. (2021) Rapid and efficient generation of myelinating human oligodendrocytes in organoids. Front. Cell. Neurosci. 15, 631548 10.3389/fncel.2021.631548 33815061 PMC8010307

[172] Madhavan M., Nevin Z.S., Shick H.E., Garrison E., Clarkson-Paredes C., Karl M. et al. Induction of myelinating oligodendrocytes in human cortical spheroids. Nat. Methods. 15, 700–706 10.1038/s41592-018-0081-4 30046099 PMC6508550

[173] Mertens J., Paquola A.C.M., Ku M., Hatch E., Böhnke L., Ladjevardi S. et al. (2015) Directly reprogrammed human neurons retain aging-associated transcriptomic signatures and reveal age-related nucleocytoplasmic defects. Cell Stem Cell. 17, 705–718 10.1016/j.stem.2015.09.001 26456686 PMC5929130

[174] Mertens J., Reid D., Lau S., Kim Y. and Gage F.H. (2018) Aging in a dish: iPSC-derived and directly induced neurons for studying brain aging and age-related neurodegenerative diseases. Annu. Rev. Genet. 52, 271–293 30208291 10.1146/annurev-genet-120417-031534 PMC6415910

[175] Xu J., Fang S., Deng S., Li H., Lin X., Huang Y. et al. (2023) Generation of neural organoids for spinal-cord regeneration via the direct reprogramming of human astrocytes. Nat. Biomed. Eng. 7, 253–269 10.1038/s41551-022-00963-6 36424465 PMC12889199

[176] Zhou-Yang L., Eichhorner S., Karbacher L., Böhnke L., Traxler L. and Mertens J. (2021) Direct conversion of human fibroblasts to induced neurons. Methods Mol. Biol. 2352, 73–96 10.1007/978-1-0716-1601-7_6 34324181

[177] Traxler L., Edenhofer F. and Mertens J. (2019) Next-generation disease modeling with direct conversion: a new path to old neurons. FEBS Lett. 593, 3316–3337 10.1002/1873-3468.13678 31715002 PMC6907729

[178] Held A., Adler M., Marques C., Reyes C.J., Kavuturu A.S., Quadros A.R.A.A. et al. (2023) iPSC motor neurons, but not other derived cell types, capture gene expression changes in postmortem sporadic ALS motor neurons. Cell Rep. 42, 113046 10.1016/j.celrep.2023.113046 37651231 PMC10622181

[179] Israel M.A., Yuan S.H., Bardy C., Reyna S.M., Mu Y., Herrera C. et al. (2012) Probing sporadic and familial Alzheimer’s disease using induced pluripotent stem cells. Nature 482, 216–220 10.1038/nature10821 22278060 PMC3338985

[180] Victor M.B., Richner M., Olsen H.E., Lee S.W., Monteys A.M., Ma C. et al. (2018) Striatal neurons directly converted from Huntington’s disease patient fibroblasts recapitulate age-associated disease phenotypes. Nat. Neurosci. 21, 341–352 10.1038/s41593-018-0075-7 29403030 PMC5857213

[181] Doege C.A. and Abeliovich A. (2014) Dementia in a Dish. Biol. Psychiatry 75, 558–564 10.1016/j.biopsych.2014.01.007 24629668

[182] Lancaster M.A., Renner M., Martin C.A., Wenzel D., Bicknell L.S., Hurles M.E. et al. (2013) Cerebral organoids model human brain development and microcephaly. Nature 501, 373–379 10.1038/nature12517 23995685 PMC3817409

[183] Lancaster M.A. and Knoblich J.A. (2014) Organogenesis in a dish: modeling development and disease using organoid technologies. Science (New York, NY). 345, 1247125 10.1126/science.1247125 25035496

[184] Lei T., Zhang X., Fu G., Luo S., Zhao Z., Deng S. et al. (2024) Advances in human cellular mechanistic understanding and drug discovery of brain organoids for neurodegenerative diseases. Ageing Res. Rev. 102, 102517–102517 10.1016/j.arr.2024.102517 39321879

[185] Bowles K.R., Silva M.C., Whitney K., Bertucci T., Berlind J.E., Lai J.D. et al. (2021) ELAVL4, splicing, and glutamatergic dysfunction precede neuron loss in MAPT mutation cerebral organoids. Cell 184, 4547.e4517–4563.e4517 10.1016/j.cell.2021.07.003 34314701 PMC8635409

[186] Zhao J., Ikezu T.C., Lu W., Macyczko J.R., Li Y., Lewis-Tuffin L.J. et al. (2023) APOE deficiency impacts neural differentiation and cholesterol biosynthesis in human iPSC-derived cerebral organoids. Stem Cell Res. Ther. 14, 214–214 10.1186/s13287-023-03444-y 37605285 PMC10441762

[187] Zhao J., Lu W., Ren Y., Fu Y., Martens Y.A., Shue F. et al. (2021) Apolipoprotein E regulates lipid metabolism and α-synuclein pathology in human iPSC-derived cerebral organoids. Acta Neuropathol. (Berl) 142, 807–825 10.1007/s00401-021-02361-9 34453582 PMC8500881

[188] Kim H., Park H.J., Choi H., Chang Y., Park H., Shin J. et al. (2019) Modeling G2019S-LRRK2 sporadic Parkinson’s disease in 3D midbrain organoids. Stem Cell Rep. 12, 518–518 10.1016/j.stemcr.2019.01.020 30799274 PMC6410341

[189] Bose A., Petsko G.A. and Studer L. (2022) Induced pluripotent stem cells: a tool for modeling Parkinson’s disease. Trends Neurosci. 45, 608–620 10.1016/j.tins.2022.05.001 35667922 PMC9576003

[190] Smits L.M., Reinhardt L., Reinhardt P., Glatza M., Monzel A.S., Stanslowsky N. et al. (2019) Modeling Parkinson’s disease in midbrain-like organoids. npj Parkinsons Dis. 5, 1–8 30963107 10.1038/s41531-019-0078-4 PMC6450999

[191] Urrestizala-Arenaza N., Cerchio S., Cavaliere F. and Magliaro C. (2024) Limitations of human brain organoids to study neurodegenerative diseases: a manual to survive. Front. Cell. Neurosci. 18, 1419526–1419526 10.3389/fncel.2024.1419526 39049825 PMC11267621

[192] Zhao H.H. and Haddad G. (2024) Brain organoid protocols and limitations. Front. Cell. Neurosci. 18, 1351734–1351734 10.3389/fncel.2024.1351734 38572070 PMC10987830

[193] Agboola O.S., Hu X., Shan Z., Wu Y. and Lei L. (2021) Brain organoid: a 3D technology for investigating cellular composition and interactions in human neurological development and disease models *in vitro*. Stem Cell Res. Therapy 12, 1–16 10.1186/s13287-021-02369-8 PMC8325286 34332630

[194] James O.G., Selvaraj B.T., Magnani D., Burr K., Connick P., Barton S.K. et al. (2021) iPSC-derived myelinoids to study myelin biology of humans. Dev. Cell. 56, 1346.e1346–1358.e1346 10.1016/j.devcel.2021.04.006 33945785 PMC8098746

[195] Sun X.Y. and Luo Z.G. (2022) Vascularizing the brain organoids. J. Mol. Cell Biol. 14, 40–40 10.1093/jmcb/mjac040 PMC9412824 35751626

[196] Sun X.Y., Ju X.C., Li Y., Zeng P.M., Wu J., Zhou Y.Y. et al. (2022) Generation of vascularized brain organoids to study neurovascular interactions. eLife 11, e76707–e76707 10.7554/eLife.76707 35506651 PMC9246368

[197] Marton R.M., Miura Y., Sloan S.A., Li Q., Revah O., Levy R.J. et al. (2019) Differentiation and maturation of oligodendrocytes in human three-dimensional neural cultures. Nat. Neurosci. 22, 484–491 10.1038/s41593-018-0316-9 30692691 PMC6788758

[198] Sabate-Soler S., Nickels S.L., Saraiva C., Berger E., Dubonyte U., Barmpa K. et al. (2022) Microglia integration into human midbrain organoids leads to increased neuronal maturation and functionality. Glia 70, 1267–1288 10.1002/glia.24167 35262217 PMC9314680

[199] Onesto M.M., Kim J.I. and Pasca S.P. (2024) Assembloid models of cell-cell interaction to study tissue and disease biology. Cell Stem Cell 31, 1563–1563 10.1016/j.stem.2024.09.017 39454582 PMC12143640

[200] Ozgun A., Lomboni D.J., Aylsworth A., Macdonald A., Staines W.A., Martina M. et al. (2024) Unraveling the assembloid: Real-time monitoring of dopaminergic neurites in an inter-organoid pathway connecting midbrain and striatal regions. Mater. Today Bio. 25, 100992–100992 10.1016/j.mtbio.2024.100992 38371467 PMC10873721

[201] Tran H.-D., Shin M.-K., Yeo X.Y., Jung S., Junaid M., Lim S.B. et al. (2025) A human striatal-midbrain assembloid model of alpha-synuclein propagation. Brain. 149, 867–883 10.1093/brain/awaf326 40919647

[202] Park J., Lee B.K., Jeong G.S., Hyun J.K., Lee C.J. and Lee S.H. (2015) Three-dimensional brain-on-a-chip with an interstitial level of flow and its application as an *in vitro* model of Alzheimer’s disease. Lab Chip 15, 141–150 10.1039/C4LC00962B 25317977

[203] Cho A.N., Jin Y., An Y., Kim J., Choi Y.S., Lee J.S. et al. (2021) Microfluidic device with brain extracellular matrix promotes structural and functional maturation of human brain organoids. Nat. Commun. 12, 1–23 10.1038/s41467-021-24775-5 34354063 PMC8342542

[204] Wang Y., Wang L., Guo Y., Zhu Y. and Qin J. (2018) Engineering stem cell-derived 3D brain organoids in a perfusable organ-on-a-chip system. RSC Adv. 8, 1677–1677 10.1039/C7RA11714K 35540867 PMC9077091

[205] Pramotton F.M., Spitz S. and Kamm R.D. (2024) Challenges and future perspectives in modeling neurodegenerative diseases using organ‐on‐a‐chip technology. Adv. Sci. 11, 2403892–2403892 10.1002/advs.202403892 38922799 PMC11348103

[206] Cai H., Ao Z., Wu Z., Song S., Mackie K. and Guo F. (2021) Intelligent acoustofluidics enabled mini-bioreactors for human brain organoids. Lab Chip 21, 2194–2194 10.1039/D1LC00145K 33955446 PMC8243411

[207] Habibey R., Rojo Arias J.E., Striebel J. and Busskamp V. (2022) Microfluidics for neuronal cell and circuit engineering. Chem. Rev. 122, 14842–14842 10.1021/acs.chemrev.2c00212 36070858 PMC9523714

[208] Platani M., Jiang H., Davidson L., Hariharan S., Doyonnas R., Lamond A.I. et al. (2025) Screening for variable drug responses using human iPSC cohorts. PloS One 20, e0323953–e0323953 10.1371/journal.pone.0323953 40445916 PMC12124524

